# Sweet Taste and Nutrient Value Subdivide Rewarding Dopaminergic Neurons in *Drosophila*

**DOI:** 10.1016/j.cub.2015.01.036

**Published:** 2015-03-16

**Authors:** Wolf Huetteroth, Emmanuel Perisse, Suewei Lin, Martín Klappenbach, Christopher Burke, Scott Waddell

**Affiliations:** 1 Centre for Neural Circuits and Behaviour, The University of Oxford, Tinsley Building, Mansfield Road, Oxford OX1 3SR, UK; 2 Zukunftskolleg, University of Konstanz, Universitätsstraße 10, 78457 Konstanz, Germany; 3 Laboratorio de Neurobiología de la Memoria, Departamento de Fisiología y Biología Molecular y Celular, IFIBYNE-CONICET, Pabellón II, Facultad de Ciencias Exactas y Naturales, Universidad de Buenos Aires, Buenos Aires C1428EGA, Argentina

## Abstract

Dopaminergic neurons provide reward learning signals in mammals and insects [1–4]. Recent work in *Drosophila* has demonstrated that water-reinforcing dopaminergic neurons are different to those for nutritious sugars [5]. Here, we tested whether the sweet taste and nutrient properties of sugar reinforcement further subdivide the fly reward system. We found that dopaminergic neurons expressing the OAMB octopamine receptor [6] specifically convey the short-term reinforcing effects of sweet taste [4]. These dopaminergic neurons project to the β′_2_ and γ_4_ regions of the mushroom body lobes. In contrast, nutrient-dependent long-term memory requires different dopaminergic neurons that project to the γ_5b_ regions, and it can be artificially reinforced by those projecting to the β lobe and adjacent α_1_ region. Surprisingly, whereas artificial implantation and expression of short-term memory occur in satiated flies, formation and expression of artificial long-term memory require flies to be hungry. These studies suggest that short-term and long-term sugar memories have different physiological constraints. They also demonstrate further functional heterogeneity within the rewarding dopaminergic neuron population.

## Highlights

• Sweet taste and nutrient value recruit different reinforcing dopaminergic neurons
• Sweetness and nutrient value separately reinforce short- and long-term memories
• Reinforcement of short-term memory is not dependent on the state of hunger
• Acquisition and retrieval of long-term memory are hunger state dependent

## Teaser

A small number of dopaminergic neurons in the fly brain are crucial for appetitive memory reinforcement. Huetteroth et al. further subdivide these rewarding neurons into those required for sweet taste reinforcement of short-term memory, nutrient-dependent long-term memory, and others that can artificially reinforce food-independent long-term memory.

## Results and Discussion

Sweet taste and nutrient value of sugars reinforce learning in *Drosophila* [7, 8]. Octopaminergic neurons specifically convey sweet taste signals [4, 9]. Blocking them impaired short-term memory (STM) reinforced by the sweet but non-nutritious arabinose. In contrast, long-term memory (LTM) formed with sweet and nutritious sucrose was unaffected. Reinforcing octopamine activates a subpopulation of dopaminergic neurons via the Ca^2+^-coupled α-adrenergic-like octopamine receptor OAMB. However, despite the evident separation of memory phases with octopamine [4, 10], manipulating dopaminergic neurons has so far impacted sweet taste and nutrient-reinforced memory [3, 4]. We therefore investigated whether octopamine dependence separates rewarding dopaminergic neurons.

**Figure 1:**
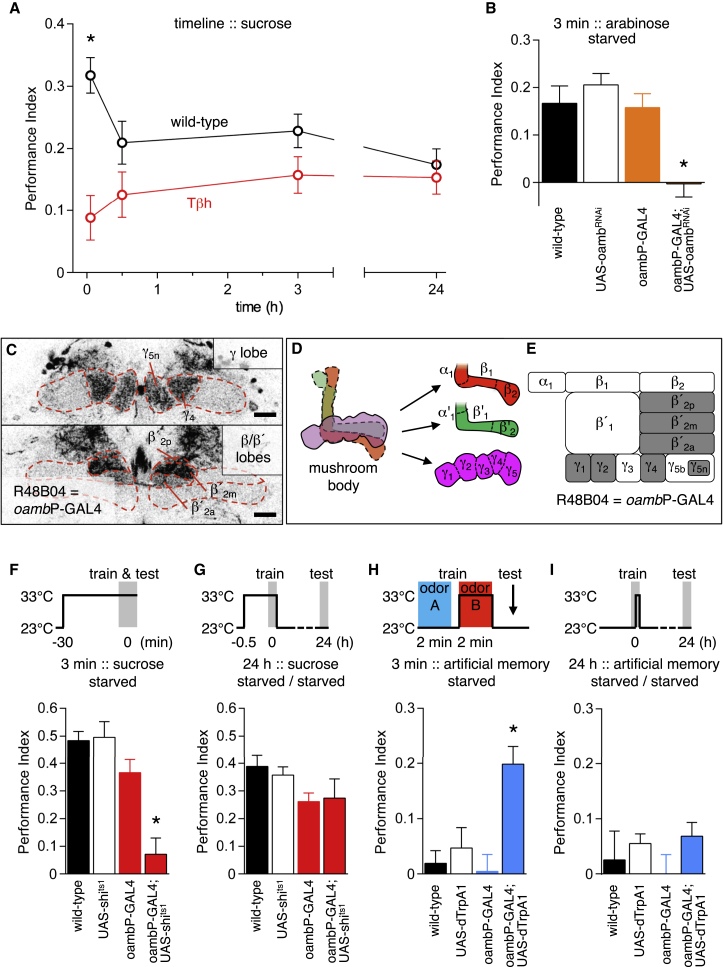
Sweet Taste Reinforces Short-Term Memory via Octopamine Signaling in *oamb*P-GAL4 Dopaminergic Neurons (A) *Tbh*^*M18*^ flies exhibit defective STM following training with concentrated sucrose (compared to wild-type, p < 0.0001, t test). Residual memory of *Tbh*^*M18*^ flies persists and is statistically indistinguishable from memory in wild-type flies 30 min, 3 hr, and 24 hr after training (all p > 0.7, t test). All n ≥ 12. (B) Hungry *oamb*P-GAL4;UAS-*oamb*^RNAi^ flies lack STM following training with arabinose (versus controls, p < 0.0001, ANOVA, n ≥ 8). (C) R48B04 (referred to as *oamb*P-GAL4) labels about 55 dopaminergic neurons that zonally innervate γ_1_, γ_2_, γ_4_, and γ_5n_ of the γ lobe and β′_2a_, β′_2m_, and β′_2p_ of the β′ lobe. 1.5-μm frontal confocal sections at the level of the γ lobe and β lobes are shown; scale bars represent 20 μm. See Figure S1A for full brain expression. (D) Schematic of the mushroom body lobes and additional zonal suborganization of the horizontal β (red), β′ (green), and γ (magenta) lobes. The β_1,_ β_2_ and β′_1_, β′_2_ border their respective α_1_ and α′_1_ subregions on the base of the vertical lobes. The exclusively horizontal γ lobe can be split into γ_1_–γ_5_ [12]. (E) Illustration of the lobe subregions, highlighting those innervated by *oamb*P-GAL4 dopaminergic neurons (gray). (F and G) Blocking *oamb*P-GAL4 neuron output with UAS-*shi*^ts1^ significantly impairs STM in starved flies trained with sucrose (p < 0.0001, ANOVA, n = 8) (F), whereas it has no effect on 24-hr LTM, as compared to controls (p > 0.1, ANOVA, n = 8) (G). (H) Pairing odor exposure with dTrpA1 activation of *oamb*P-GAL4 neurons forms STM that is significantly different to control flies (p < 0.0002, ANOVA, n ≥ 9). (I) Artificially implanted *oamb*P-GAL4 memory is labile. Performance of hungry *oamb*P-GAL4; UAS-*dTrpA1* flies is not statistically different to that of controls at 24 hr (p > 0.1, ANOVA, n ≥ 10).

*Tbh*^*M18*^ mutant flies, lacking octopamine, cannot form STM reinforced with 2 M sucrose [11]. However, a persistent memory slowly emerges after training *Tbh*^*M18*^ flies with odor and 1 M sucrose [10], suggesting that nutrient-dependent LTM is likely to be formed in parallel and independent of appetitive STM. Since nutrient-dependent memory can guide behavior as quickly as 2 min after training [7], we first determined whether nutrient memory could be observed in wild-type and *Tbh*^*M18*^ flies trained with saturated sucrose, ∼5.8 M (Figure 1A). Strikingly, this analysis revealed performance at all times in *Tbh*^*M18*^ flies that was statistically different to wild-type immediately after training but indistinguishable from wild-type 30 min, 3 hr, and 24 hr after training. These data are consistent with *Tbh*^*M18*^ flies only lacking sweet-taste-reinforced STM [4, 10]. Moreover, they demonstrate that nutrient-dependent (octopamine-independent) memory is observable immediately after training with high sucrose concentrations.

Prior knowledge that octopamine activates rewarding dopaminergic neurons through the OAMB receptor [4] led us to identify R48B04-GAL4 in the FlyLight collection [13]. R48B04-GAL4 is driven by a promoter fragment from the *oamb* gene (although we acknowledge that this reagent is unlikely to label all *oamb*-expressing neurons, from here on, we will refer to it as *oamb*P-GAL4). We verified the relevance of *oamb*P-GAL4 neurons by knocking down OAMB expression with UAS-*oamb*^RNAi^ [4, 14]. As expected, these flies completely lacked STM when trained with the sweet and non-nutritious sugar arabinose (Figure 1B). The memory defect was more pronounced than when OAMB was knocked down in dopaminergic neurons with 0104-GAL4 [4], suggesting *oamb*P-GAL4 may more accurately label octopamine-responsive dopaminergic neurons than 0104-GAL4 (Figures S1A–S1E). Our initial examination of *oamb*P-GAL4 revealed expression in approximately 55 rewarding dopaminergic neurons (and ∼12 tyrosine hydroxylase (TH)-negative neurons) in the protocerebral anterior medial (PAM) cell cluster that innervate the horizontal mushroom body lobes (Figures 1C–1E, S1A, S1C, and S1D; [5]).

We next tested the contribution of *oambP*-GAL4 neurons to saturated sucrose-reinforced memory by blocking their output using the dominant temperature-sensitive UAS-*shibire*^ts1^ (UAS-*shi*^ts1^) transgene [15]. Blocking *oamb*P-GAL4 neurons significantly impaired STM (Figure 1F). However, LTM performance of *oamb*P-GAL4;UAS-*shi*^ts1^ flies was indistinguishable from controls, demonstrating a specific loss of STM (Figure 1G), consistent with *Tbh*^*M18*^ flies trained with sucrose (Figure 1A).

We also tested a reinforcing role of *oambP*-GAL4 neurons by pairing their activation, using UAS-*dTrpA1*, with odor presentation (Figures 1H and 1I). The *dTrpA1*-encoded transient receptor potential (TRP) channel conducts Ca^2+^ and depolarizes neurons when temperature exceeds 25°C [16]. This protocol implanted STM that was statistically different from all controls in both starved and fed flies (Figures 1H and S1F). However, implanted memory did not persist. Performance of *oamb*P-GAL4;UAS-*dTrpA1* flies was indistinguishable from controls 24 hr after training (Figure 1I). Taken together, these data suggest that *oamb*P-GAL4 dopaminergic neurons specifically convey octopamine-dependent and hunger-state-independent sweet taste reinforcement, whereas other rewarding dopaminergic neurons contribute nutrient value signals.

**Figure 2:**
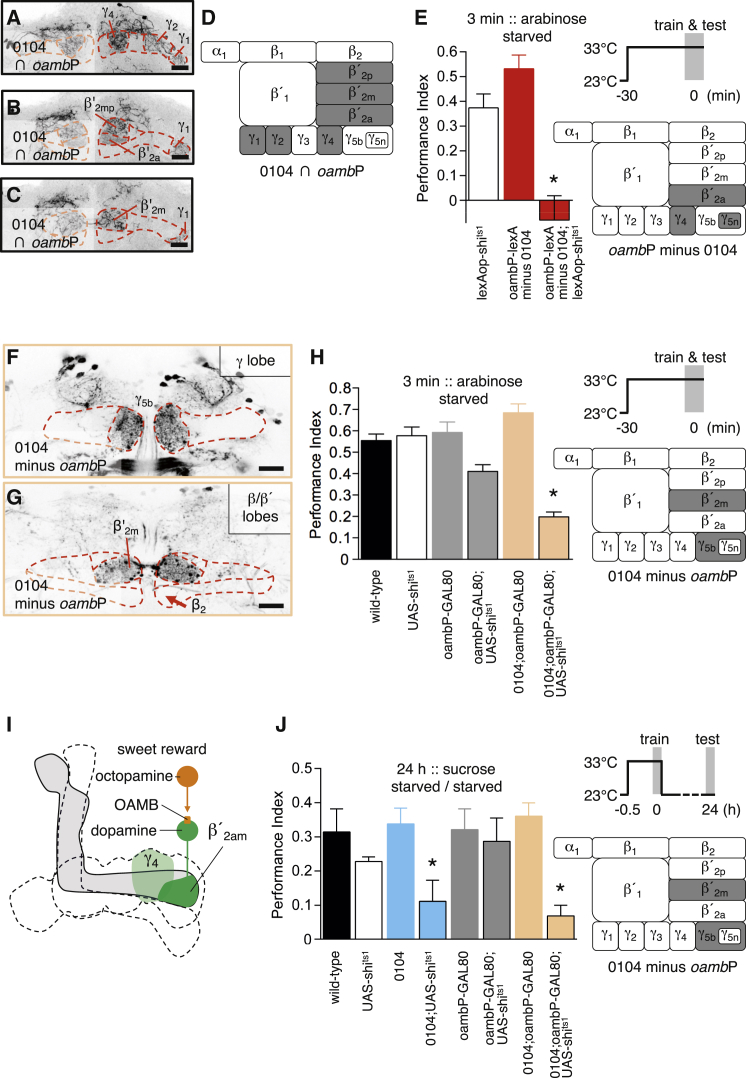
Short-Term Memory Reinforcement by Sweet Taste Requires β′_2_ and γ_4_ Dopaminergic Neurons (A–C) Positive intersection between 0104-GAL4 and *oamb*P-lexA with lexAop-FLP,tub>GAL80>STOP and UAS-mCD8::GFP labels about 20 neurons. (A) Projection of 20 1-μm confocal sections at the level of the γ lobe reveals processes in γ_1_, γ_2_, and γ_4_. (B) Projection of ten 1-μm confocal sections at the level of the β and β′ lobes shows innervation in β′_2a_ and β′_2mp_ and some posterior γ_1_ from the same cell type as in (A). (C) Projection of ten 1-μm confocal sections of the β′ lobe reveals shared 0104-GAL4 and *oamb*P-LexA innervation of β′_2m_. Scale bars of (A)–(C) represent 20 μm. (D) Illustration summarizing the zones of the mushroom body innervated by neurons common to 0104-GAL4 and *oamb*P-lexA. (E) *oamb*P neurons not labeled by 0104 are required for STM formation with arabinose. Performance of starved *oamb*P-LexA/lexAop-*shi*^ts1^;0104-GAL4/UAS-*lexA*^RNAi^ flies is significantly different to controls (p < 0.0001, ANOVA, n = 8). See permissive temperature control in Figure S1G. Illustration demonstrates dopaminergic neurons unique to *oamb*P-lexA/*oamb*P-GAL4. (F and G) Removing *oamb*P neurons from 0104 (*oamb*P-GAL80/20xUAS-6xGFP;0104-GAL4) leaves expression in about 15 neurons innervating γ_5b_ (F) and β′_2m_ (G) neurons. Projection of five 2-μm confocal sections covering the γ lobe (F) and the β/β′ lobe (G). *oamb*P-GAL80 also unexpectedly suppresses expression in the β_2_ (red arrow), which is otherwise labeled by 0104-GAL4 (Figure S1B). Scale bars represent 20 μm. (H) Blocking output from γ_5b_ and β′_2m_ dopaminergic neurons, shown in illustration, during arabinose-reinforced training impairs STM. Performance of *oamb*P-GAL80;0104-GAL4/UAS-*shi*^ts1^ flies was significantly different to controls (p < 0.0001, ANOVA, n = 8). See permissive temperature control in Figure S1H. (I) Summary of STM formation. Sweet taste engages octopaminergic neurons. Octopamine acts through the OAMB receptor to activate rewarding dopaminergic neurons that innervate β′_2am_ (and γ_4_) regions of the mushroom body. (J) Blocking output from the β′_2m_ and γ_5b_ subset of 0104-GAL4 neurons during sucrose-reinforced training impairs 24-hr LTM. Performance of 0104-GAL4/UAS-*shi*^ts1^ and *oamb*P-GAL80;0104-GAL4/UAS-*shi*^ts1^ flies was significantly different to controls (p < 0.0002, ANOVA, n = 5-12). See permissive temperature control in Figure S2B.

0104-GAL4 labels octopamine-responsive dopaminergic neurons and some required for nutrient reinforcement [4]. We reasoned that intersecting 0104-GAL4 and *oamb*P-GAL4 would separate sweet and nutrient reinforcement. Common neurons in 0104-GAL4 and *oamb*P-GAL4 can be visualized by combining R48B04-LexA (i.e., *oamb*P-LexA, which expresses in *oamb*P-GAL4 dopaminergic neurons; Figure S1C) with 0104-GAL4-driven UAS>STOP>GFP (where > represents a FLP-recombinase target sequence) and lexAop-*FLP*. In these flies, GFP labels 10–20 dopaminergic neurons innervating the anterior, median, and posterior β′_2_ (β′_2amp_) and γ_4_ zones of the mushroom body, in addition to a new class of TH-negative neurons that connect γ_1_, γ_2_, and γ_4_ (Figures 2A–2D). Both *oamb*P-GAL4 and 0104-GAL4 contain γ_5_-innervating neurons (Figures 1C and S1A–S1E), but the positive intersection does not label them, suggesting that each GAL4 includes unique γ_5_ neurons: γ_5narrow_ (γ_5n_) in *oamb*P-GAL4 (Figures 1C, S1A, S1C, and S1D) and γ_5broad_ (γ_5b_) in 0104-GAL4 (Figures S1B and S1E).

To assess the role of subsets of *oamb*P-labeled neurons, we removed expression in 0104 neurons by combining 0104-GAL4 with UAS-*lexA*^RNAi^,*oamb*P-LexA flies and a lexAop-*shi*^*ts*1^ transgene, thereby restricting expression to dopaminergic neurons innervating β′_2a,_ γ_4_, and γ_5n_ [5]. These flies exhibited no STM following training with sweet-only arabinose at restrictive 33°C (Figure 2E). No significant defect was evident at permissive 23°C (Figure S1G). We also constructed 0104-GAL4;*oambP*-GAL80 flies in which GAL80 inhibits GAL4-driven gene expression [17] resulting in expression being restricted to β′_2m_ and γ_5b_ 0104 neurons (Figures 2F–2H). These flies also displayed defective STM following conditioning at restrictive 33°C with arabinose (Figure 2H), while no significant defect was evident at permissive 23°C (Figure S1H). Since blocking γ_5b_ and γ_5n_ neurons with R15A04-GAL4 does not impair STM (Figure S2A), we conclude that sweet taste reinforcement is conveyed by octopaminergic signaling through the OAMB receptor in dopaminergic neurons that innervate the β′_2am_ and γ_4_ zones of the mushroom body (Figure 2I).

0104-GAL4 also includes neurons required for nutrient-dependent LTM, which are not in *oamb*P-GAL4. Indeed, blocking 0104-GAL4;*oambP*-GAL80 neurons with UAS-*shi*^ts1^ revealed a significant LTM defect (Figure 2J). No defects were apparent at the permissive temperature (Figure S2B). These data indicate that dopaminergic neurons in β′_2m_ and/or γ_5b_ are required for nutrient-dependent LTM formation.

**Figure 3:**
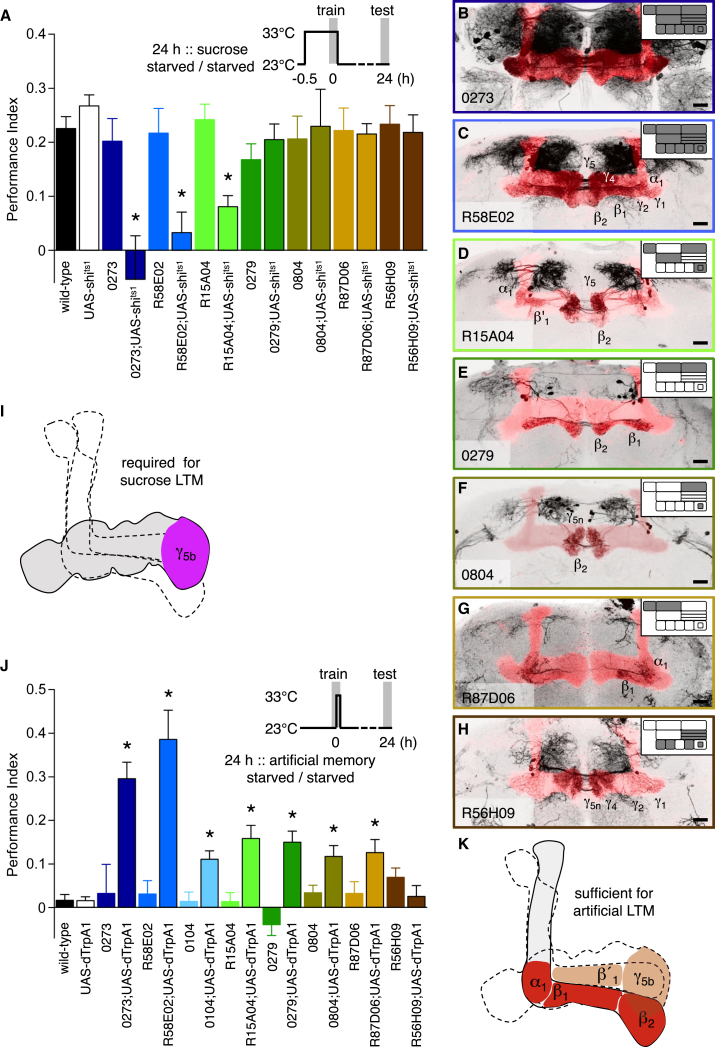
Dopaminergic Neurons Required for Nutrient-Dependent LTM Differ from Those that Can Artificially Implant LTM (A) Blocking output from 0273, R58E02, and R15A04 neurons during sucrose training significantly disrupted LTM in starved flies (all p < 0.001, ANOVA, n ≥ 9). See permissive temperature control in Figure S2C. LTM was not statistically impaired by blocking 0279, 0804, R87D06, or R56H09 neurons (p > 0.7, ANOVA, n ≥ 8). (B–H) Mushroom body lobe (red) innervation of 0273-GAL4 (B) [4], R58E02-GAL4 (C) [3], R15A04-GAL4 (D), 0279-GAL4 (E) [18], 0804-GAL4 (F), R87D06-GAL4 (G), and R56H09-GAL4 (H) revealed with UAS-mCD8::GFP. The mushroom body (red) is labeled in each brain with 247-lexA::VP16-driven lexAop-rCD2::mRFP [19]. Scale bars represent 20 μm. Zonal innervation of each line is shown in the corresponding inset illustration. (I) Summary. Dopaminergic neurons innervating γ_5b_ are essential to reinforce nutrient-dependent LTM. (J) Pairing odor with dTrpA1 activation of 0273, R58E02, 0104, 0279, R15A04, 0804, and R87D06 dopaminergic neurons forms significant LTM (all p < 0.001, ANOVA, n ≥ 6). No significant memory was formed in R56H09;UAS-*dTrpA1* flies (p > 0.3, ANOVA, n ≥ 10). All flies were food deprived before and after training. (K) Summary. Dopaminergic neurons innervating α_1_, β_1_, or β_2_ (and perhaps β′_1_ and γ_5b_) are sufficient to form LTM.

We next visually screened for GAL4 lines with expression in PAM dopaminergic neurons that innervate the horizontal mushroom body lobes. We used these and three established PAM lines [3, 4, 18] to express UAS-*shi*^ts1^ and tested LTM following sucrose-reinforced learning. Blocking 0273, R58E02, or R15A04 neurons during training significantly impaired LTM performance compared to the relevant controls (Figure 3A). In contrast, blocking 0279, 0804, R87D06, or R56H09 neurons did not. No significant defects were apparent when R15A04;UAS-*shi*^ts1^, R58E02;UAS-*shi*^ts1^, or 0273;UAS-*shi*^ts1^ flies were trained and tested at permissive 23°C (Figure S2C).

**Figure 4:**
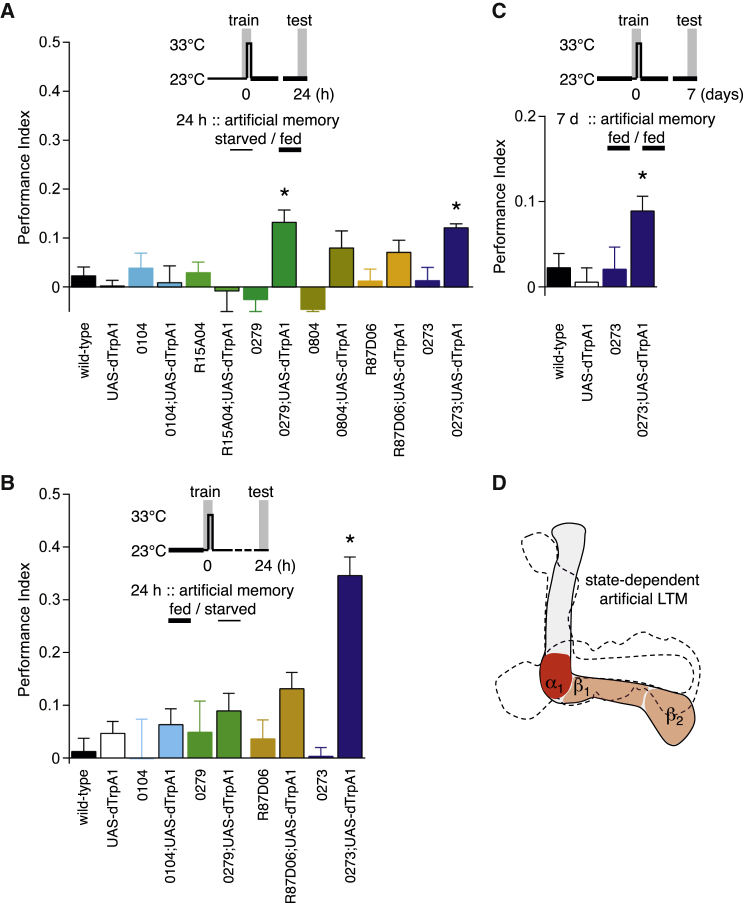
Distinct Dopaminergic Neurons Form Hunger-State-Dependent and Hunger-Independent LTM (A) Feeding after training suppresses artificially implanted LTM performance in 0104, R15A04, 0804, and R87D06 flies expressing UAS-*dTrpA1* (all p > 0.1, ANOVA, n ≥ 9). Statistically significant memory remained after feeding in 0279;UAS-*dTrpA1* and 0273;UAS-*dTrpA1* flies (p < 0.0001, ANOVA, n ≥ 8). No other group showed performance that was statistically different to their respective fed controls (p > 0.08, ANOVA, n ≥ 9). (B) Significant LTM could not be formed when odor was paired with UAS-*dTrpA1*-driven activation of 0104, 0279, or R87D06 neurons in food-satiated flies, despite 24 hr of food deprivation after training (p > 0.2, ANOVA, n ≥ 6). In contrast, significant memory was formed in food-satiated 0273;UAS-*dTrpA1* flies (p < 0.0001, ANOVA, n ≥ 6). (C) Measurable artificial LTM remained in 0273;UAS-*dTrpA1* flies following 7 days of ad libitum feeding after training (p = 0.001, ANOVA, n ≥ 6). (D) Summary. Formation and retrieval of LTM by dopaminergic neurons innervating α_1_, β_1_, and β_2_ are sensitive to feeding before and/or after training.

0273 and R58E02 label ∼130 and ∼90 dopaminergic neurons, respectively, that broadly innervate the horizontal lobes (Figures 3B and 3C; [3, 4]). R15A04 expresses in ∼26 dopaminergic neurons projecting to α_1_, β′_1_, β_2_, γ_5b_, and γ_5n_ (Figures 3D, S2D, and S3K). These overlap with 0104 in β_2_ and γ_5b_. Ineffective GAL4 lines further refine necessary nutrient-reinforcing neurons (Figures S3 and S4A–S4C; Table S1). Briefly, 0279-GAL4 dopaminergic neurons innervate β_1_ and β_2_ (Figure 3E; [18]). 0804-GAL4 innervate β_2_ and γ_5n_ (Figures 3F, S2E, and S3L), R87D06-GAL4 project to α_1_ and β_1_ (Figures 3G, S2F, and S3M), and R56H09-GAL4 innervate β′_2m_ and γ_5n_ (Figures 3H, S2G, and S3J). These negative data indicate that β_2_ and γ_5n_ innervation is dispensable (Figures 3A, 3E–3H, and S4C). Therefore, we conclude that nutrient reinforcement requires dopaminergic neurons innervating γ_5b_ (Figure 3I).

Artificially activating large groups of 0273 or R58E02 dopaminergic neurons paired with odor formed robust appetitive memory [3, 4]. We therefore tested for subsets that were sufficient to reinforce LTM (Figure 3J). We combined each GAL4 with UAS-*dTrpA1* and paired dTrpA1-activating 33°C with odor. Surprisingly, 0273, R58E02, 0104, 0279, R15A04, 0804, and R87D06 produced LTM performance that was statistically different to their relevant control flies, whereas R56H09 did not (Figure 3J). Notably, 0279, 0804, and R87D06 neurons, which were not required for sucrose LTM, reinforced artificial 24-hr memory. These and all other LTM-competent lines (0273, R58E02, 0104, R15A04) include dopaminergic innervation of the β lobe or adjacent α_1_ lobe (Figures 3B–3G, S1B, S1E, and S4C; Table S1), whereas those that cannot implant LTM lack projections to these regions (R48B04, R56H09; Figures 1C–1E, 3H, S1A, S1C, S1D, S3J, and S4C; Table S1). Therefore, we conclude that artificial LTM can be formed by dopaminergic neurons innervating α_1_, β_1_, and β_2_ (Figure 3K). Furthermore, removal of STM-reinforcing *oamb*P-LexA neurons (Figures 1H and 1I) from 0804 (Figure S4A) leaves expression in only two neurons innervating β_2_ (Figure S4B), suggesting that these alone may provide sufficient reinforcement for appetitive LTM. Such localization of lasting reinforcement is consistent with the importance of αβ neurons for LTM and its retrieval [20–23].

Given the discordance between the γ_5b_ neurons required for sucrose LTM and those targeting α_1_, β_1_, and β_2_ that can reinforce persistent memory, we tested the food relevance of implanted memories. Expression of sugar-reinforced memory, but not water-reinforced memory, can be suppressed by feeding flies after training [5]. Feeding suppressed LTM performance of 0104;UAS-*dTrpA1*, R15A04;UAS-*dTrpA1*, 0804;UAS-*dTrpA1*, and R87D06;UAS-*dTrpA1* flies to levels that were statistically indistinguishable from their respective fed controls (Figure 4A). In contrast, significant performance remained in 0279;UAS-*dTrpA1* and 0273;UAS-*dTrpA1* flies. Therefore, memory reinforced in the β and adjacent α_1_ regions by 0104, R15A04, 0804, and R87D06 neural activation mimics sucrose-reinforced memory, whereas 0279-implanted memory has different properties.

Although flies ordinarily need to be hungry to form sugar-reinforced appetitive memory, prior experiments and those here demonstrate that appetitive STM can be formed in fed flies by pairing octopaminergic or dopaminergic neuron activation with odor presentation (Figure S1F; [3, 4]). We therefore tested whether nutrient-dependent LTM could also be formed artificially in food-satiated flies. We analyzed food-relevant 0104-formed and R87D06-formed memory and non-food satiable 0279-formed and 0273-formed memory in parallel. Strikingly, 0104, R87D06, and 0279 activation did not form LTM in satiated flies, whereas 0273;UAS-*dTrpA1* flies exhibited robust LTM (Figure 4B), which was even evident following 7 days of ad libitum feeding after training (Figure 4C). Satiety therefore also constrains the artificial formation of appetitive LTM. We speculate that some 0273 dopaminergic neurons represent rewarding events other than food.

Taken with prior studies [4, 7, 10], results here demonstrate that the sweet taste and nutrient properties of sugars are independently processed and reinforce memories of different duration. Sweet taste is transduced through octopaminergic neurons whose released octopamine, via the OAMB receptor, activates dopaminergic neurons that project to the β′_2am_ and γ_4_ regions of the mushroom body. Octopaminergic reinforcement also modulates the state dependence of STM via the OCTβ2R receptor that is required in the dopaminergic MB-MP1 neurons [4].

Nutrient-dependent LTM does not involve octopamine [4, 10] or sweet-taste-reinforcing dopaminergic neurons. Nutrient reinforcement instead requires dopaminergic neurons innervating γ_5b_ of the mushroom body, whereas those going to β_1_, β_2_, and the adjacent α_1_ region are sufficient. More work will be required to understand this distributed process, which apparently has an immediate and delayed dynamic (Figure S4D; [7, 10]).

Whereas formation and expression of sweet-taste-reinforced STM is insensitive to satiety state, artificial formation and expression of nutrient-relevant memory require flies to be hungry. Even direct stimulation of the relevant rewarding dopaminergic neurons cannot implant appetitive LTM in food-satiated flies. These experiments suggest that hunger establishes an internal state that permits the nutrient-reinforcing signals to be effective. It will be interesting to understand what the permissive state involves and where it is required. Others have previously described a role for CRTC in enabling hunger-dependent LTM in the fly [24] and promoting persistent memory in the mouse [25]. It therefore seems plausible that such a mechanism might be required in the mushroom body neurons to permit nutrient-dependent reinforcement.

## Experimental Procedures

### Fly Strains

Fly stocks were raised on standard cornmeal food at 25°C and 50%–60% relative humidity. The wild-type *Drosophila* strain used in this study is Canton-S. The *Tbh*^*M18*^ mutant is described [26]. The UAS-mCD8::GFP, the 20xUAS-6xGFP, and the 247-lexA,lexAop-RFP flies are described [17, 19, 27]. The UAS-*oamb*^RNAi^ (strain number 2861GD) was obtained from the Vienna Drosophila Resource Center (VDRC) [14]. The UAS-*shi*^ts1^, on the first and third chromosome, and UAS-*dTrpA1* transgenic strains are described [15, 16]. The R48B04, R15A04, R87D06, and R56H09 flies [13] were obtained from Bloomington. The R58E02-LexA, R58E02-GAL80, 0104, 0273, and 0279 flies are described [3, 4, 18]. The 0804 fly strain, more correctly named PBac(IT.GAL4)0804, was generated and initially characterized by Marion Sillies and Daryl Gohl as part of the InSITE collection [28]. The R48B04-LexA, R15A04-GAL80, and UAS-*lexA*^RNAi^ flies are described [5]. The R48B04-GAL80 construct was made by inserting the enhancer fragment of R48B04-GAL4 from the Janelia Farm Research Campus FlyLight database [13] into the pBPGAL80Uw-6 vector (Addgene plasmid 26236). The R48B04-GAL80 fly strain was made commercially (BestGene) by site-specific insertion into the attP40 landing site. The UAS>STOP>GFP, lexAop-FLP, lexAop-GAL80, and *TH*-GAL80 strains are those employed in [29–31]. UAS-DenMark and UAS-DSyd1::GFP are those in [32] and [33].

To generate R48B04;UAS-*oamb*^RNAi^ flies, we crossed homozygous UAS-*oamb*^RNAi^ males to homozygous R48B04 females. R48B04/+ control flies were generated by crossing R48B04 females to wild-type males. Heterozygote UAS-*oamb*^RNAi^/+ controls were generated by crossing UAS-*oamb*^RNAi^ males to wild-type females. We generated flies expressing *shi*^ts1^ in subsets of dopaminergic neurons by crossing UAS-*shi*^ts1^ females to homozygous R48B04, 0104, R48B04-GAL80;0104, R48B04-LexA;0104, 0273, R58E02, R15A04, 0279, R87D06, or R56H09 males. 0804 resides on the X chromosome; therefore, 0804 females were crossed to UAS-*shi*^ts1^ males. Heterozygote UAS-*shi*^ts1^/+ controls were generated by crossing UAS-*shi*^ts1^ females to wild-type males. Heterozygote GAL4/+ controls were generated by crossing GAL4 males to wild-type females. We generated flies expressing *dTrpA1* in R48B04, 0273, R58E02, 0104, 0279, R15A04, R87D06, or R56H09 neurons by crossing UAS-*dTrpA1* females to homozygous R48B04, 0273, R58E02, 0104, 0279, R15A04, R87D06, or R56H09 males. Homozygous 0804 females were crossed to UAS-*dTrpA1* males. Heterozygote UAS-*dTrpA1*/+ controls were generated by crossing UAS-*dTrpA1* females to wild-type males; heterozygote GAL4/+ controls were generated by crossing GAL4 males to wild-type females and vice versa for both controls in case of 0804.

### Behavior Experiments

Appetitive memory was assayed as described [22] with the following modifications. Mixed sex populations of 4- to 8-day-old flies raised at 25°C were tested together in all behavior experiments. Before training, groups of ∼100 flies were food deprived for 18–22 hr in a 25-ml vial containing 1% agar and a 20 × 60 mm piece of filter paper. Training was performed with either saturated sucrose or 3 M arabinose as unconditioned stimulus. The odors used were 3-octanol (Sigma) and 4-methylcyclohexanol (Sigma) at 1:1,000 in mineral oil. Artificial memory implantation experiments using UAS-*dTrpA1*-mediated neural activation were performed as described [4]. Briefly, 8- to 11-day-old flies raised at 20°C were either kept in food vials or starved for 18–22 hr before training. Flies were presented with one odor at the permissive 23°C for 2 min in filter paper-lined tubes and were then transferred into a prewarmed filter paper-lined tube and immediately presented with a second odor at dTrpA1-channel activating 33°C for 2 min. Flies were then returned to 23°C and tested for immediate memory. To assay 24-hr memory, we transferred trained flies into either food vials or food deprivation vials until testing. For 7-day memory experiments, fed flies were trained and immediately transferred into food vials until memory testing after 7 days. Memory performance was assayed by allowing the flies 2 min to choose between the odors presented during training. Performance index (PI) was calculated as the number of flies approaching (appetitive memory) the conditioned odor minus the number of flies going to the unconditioned odor divided by the total number of flies in the experiment. A single PI value is the average score from flies of the identical genotype tested with the reciprocal combination of conditioned and unconditioned odor. Statistical analyses were performed using PRISM (GraphPad Software). Overall ANOVA was followed by planned pairwise comparisons between the relevant groups with a Tukey honestly significant difference (HSD) post hoc test. All experiments are n ≥ 8 unless stated otherwise.

### Imaging

To visualize native GFP or mRFP, we collected adult flies 2–11 days after eclosion, and brains were dissected in ice-cold 4% paraformaldehyde solution in PBS (1.86 mM NaH_2_PO_4_, 8.41 mM Na_2_HPO_4_, and 175 mM NaCl) and fixed for an additional 60 min at room temperature [19]. Samples were then washed 3 × 10 min with PBS containing 0.1% Triton X-100 (PBT) and 2 × 10 min in PBS before mounting in Vectashield (Vector Labs). Imaging of frontal brain views was performed on a Leica TCS SP5 X and a Zeiss LSM 510. The resolution of the image stacks were 1024 × 1024 with 0.5–1.5-μm step size and a frame average of 4. Images were processed in AMIRA 5.3 (Mercury Systems) and Fiji. The immunostaining against TH and GFP was performed as previously described [4].

## Supplemental Information

Document S1. Figures S1–S4 and Table S1

Document S2. Article plus Supplemental Information
