# Supplementary material for: Sweet Taste and Nutrient Value Subdivide Rewarding Dopaminergic Neurons in Drosophila
Source: Curr Biol. 2015 Mar 16;25(6):751–8. doi: 10.1016/j.cub.2015.01.036 (PMC4372253; doi:10.1016/j.cub.2015.01.036)
Supplement: Document S1. Figures S1–S4 and Table S1 [file mmc1.pdf]

Current Biology

Supplemental Information

Sweet Taste and Nutrient Value Subdivide Rewarding Dopaminergic Neurons in *Drosophila*

Wolf Huetteroth, Emmanuel Perisse, Suewei Lin, Martín Klappenbach, Christopher Burke, and Scott Waddell

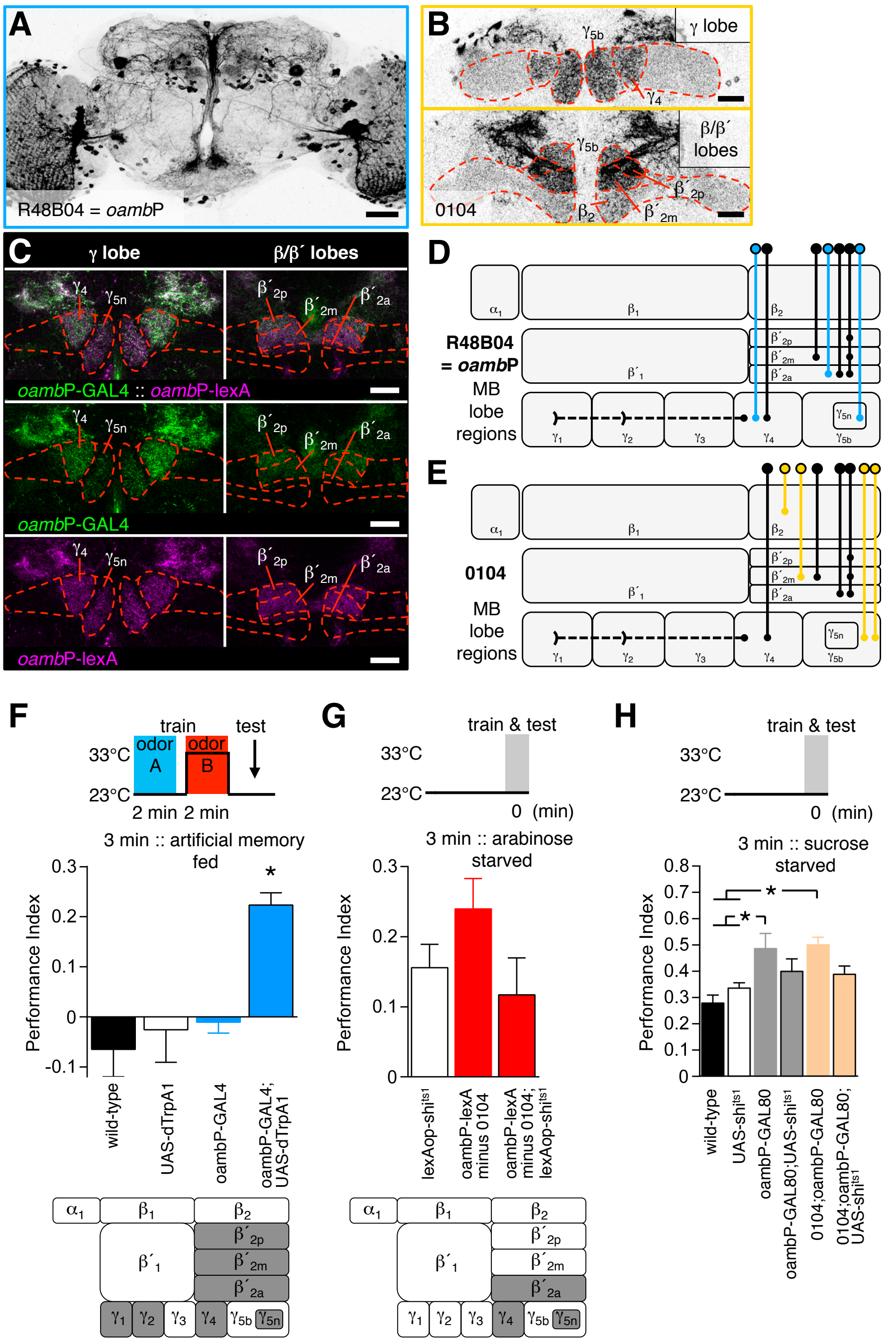

Figure S1. R48B04 (*oamb*P-GAL4, -LexA) and 0104 express in common and unique neurons. Expression data and control experiments related to Figure 1. **(A)** Projection view of a confocal stack of a brain from a *oamb*P-GAL4;UAS-mCD8::GFP fly. Scale bar 50 μm. **(B)** Individual confocal sections of 1.5 μm from a 0104-GAL4;UAS-mCD8::GFP brain at the level of the γ or β and β´ lobes, scale bar 20 μm. 0104-GAL4 driven UAS-mCD8::GFP labels ~55 mostly dopaminergic PAM cells [4]. The pattern largely overlaps with that of *oamb*P-GAL4 (Fig. 2A-D), but

0104-GAL4 labels fewer neurons innervating γ_4 and β'_{2a}. 0104-GAL4 also labels neurons innervating β'_{2m} and $\beta_{2,}$ plus the γ_{5b} cell type that broadly innervates γ_5. Detail provided in Fig. 2F, G and S3C and is summarized in Fig. S1E. **(C)** *oamb*P-GAL4 driven UAS-mCD8::GFP (green) and *oamb*P-LexA driven lexAop-rCD2::RFP (magenta) label the same subset of about 70 neurons within the PAM cluster. Individual confocal sections of 0.4 µm at the level of the γ or β/β′ lobes, scale bar 20 µm. Individual channels and the merge are shown. **(D)** Pictorial of the horizontal lobe innervation by *oamb*P-GAL4 neurons. Blue cells are exclusive to *oamb*P-GAL4, whereas black cells are common to *oamb*P-GAL4 and 0104. The cell type that connects γ_1, γ_2 and γ_4 is previously undescribed and not TH-labeled, so is unlikely to be dopaminergic. **(E)** Schematic of the horizontal lobe innervation by 0104-GAL4 neurons. Yellow cells are exclusive to 0104-GAL4, whereas black cells are common to *oamb*P-GAL4 and 0104-GAL4. **(F)** Pairing odor exposure with UAS-*dTrpA1* activation of *oamb*P-GAL4 neurons in fed flies forms appetitive STM that is significantly different to controls 3 min after training ($p < 0.003$, ANOVA, $n \geq 6$). **(G)** Permissive temperature control for Fig. 2E. STM performance of *oamb*P-LexA/lexAop-*shi*^{ts1};0104/UAS-*lexA*^{RNAi} flies was robust and indistinguishable from that of controls ($p > 0.14$, ANOVA, $n = 12$) when hungry flies were trained with arabinose at permissive 23˚C. **(H)** No significant differences were apparent in STM performance when starved *oamb*P-GAL80;0104-GAL4/UAS-*shi*^{ts1} flies were trained and tested at permissive 23°C (compared to controls, $p > 0.16$, ANOVA, $n = 8$). There is, however, a significant difference between wild type and UAS-*shi*^{ts1} and *oamb*P-GAL80;0104 controls ($p < 0.03$, ANOVA, $n = 8$).

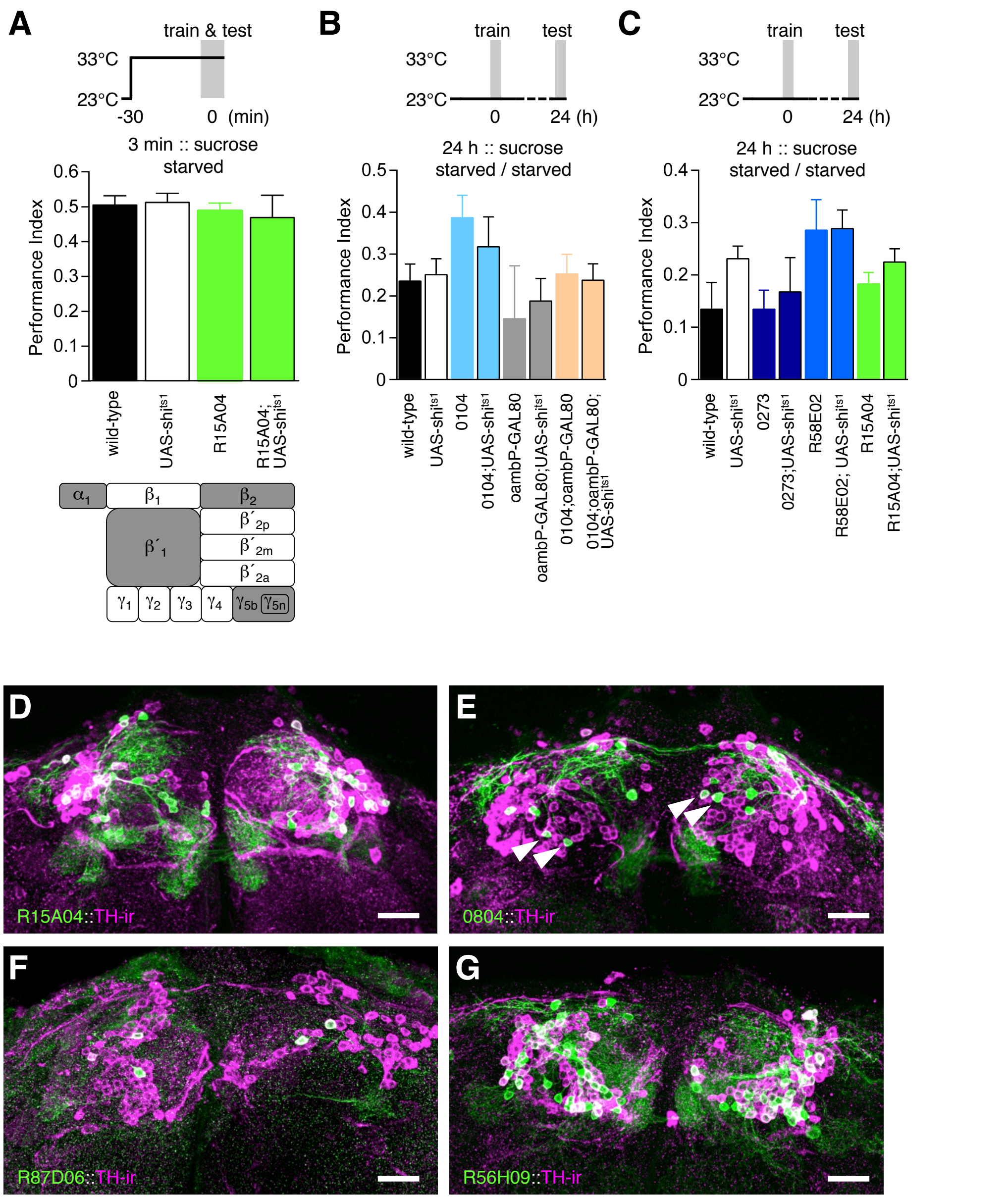

Figure S2. Permissive temperature control experiments and verification of dopaminergic neuron identity for key GAL4 lines used in Figure 2.

(A) Blocking R15A04 neurons with UAS-*shi*^{ts1} expression does not impair STM performance ($p > 0.9$, ANOVA, $n \geq 6$). **(B)** No LTM impairments were evident in starved sucrose-trained 0104-GAL4/UAS-*shi*^{ts1} or *oamb*P-GAL80;0104-GAL4/UAS-*shi*^{ts1} flies when trained and tested at permissive 23˚C ($p > 0.5$, ANOVA, n = 6-8). **(C)** At permissive 23˚C flies expressing UAS-*shi*^{ts1} with 0273, R58E02, or R15A04-GAL4 do not reveal a significant LTM defect following training with sucrose ($p > 0.4$, ANOVA, n = 5-10). **(D)** R15A04 labels ~26 tyrosine hydroxylase (TH)-positive

dopaminergic neurons and ~4 TH-negative neurons in the PAM cluster. TH-ir, anti-TH immunoreactive **(E)** Two of the ~8 neurons labeled by 0804-GAL4 are TH-positive PAM dopaminergic neurons (arrowheads). **(F)** R87D06 labels ~4-8 TH-positive PAM dopaminergic neurons. **(G)** R56H09 labels ~54 neurons in PAM. 34 are TH-positive dopaminergic neurons and the other 20 are non-dopaminergic neurons, representing a new cell type connecting γ_1, γ_2 and γ_4. Scale bars, 20 μm in D-G.

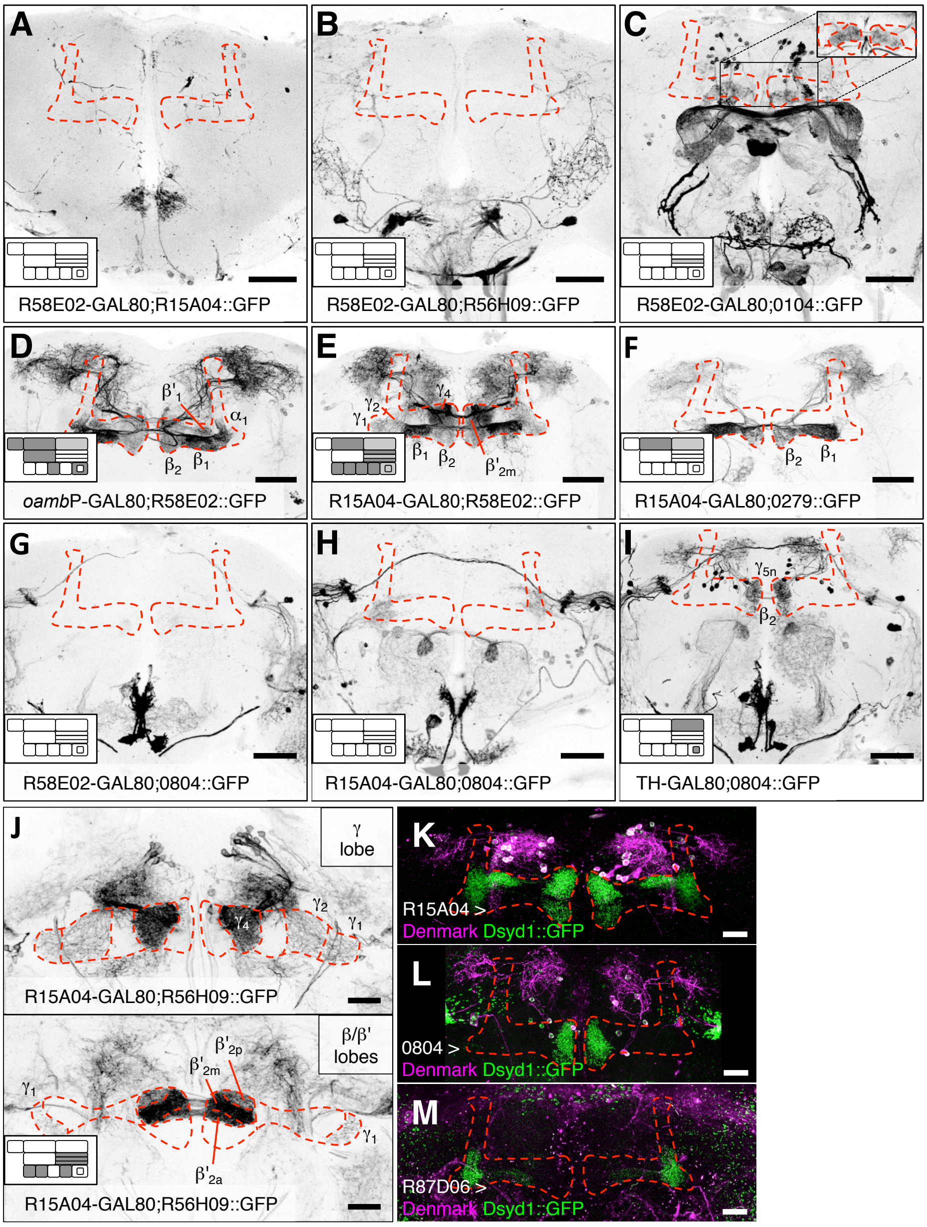

Figure S3. Genetic intersection reveals the relatedness of the dopaminergic neuron-expressing GAL4 lines used in Figure 3 and 4.

(A) R58E02-GAL80 suppresses expression in all R15A04-GAL4 labeled PAM neurons. **(B)** R58E02-GAL80 inhibits expression in R56H09-GAL4 labeled PAM neurons. **(C)** Weak expression remains in ~15 neurons that innervate β'_{2m} region when R58E02-GAL80 is combined with 0104-GAL4. Inset shows a 10 μm sub-projection at the level of the β'_{2} lobe. Scale bar, 50 μm and applies to A-C. **(D)**

*oamb*P-GAL80 combined with R58E02-GAL4;UAS-mCD8::GFP retains expression in neurons innervating the α_1, β'_1, β'_2, β_1, β_2, γ_3 and γ_{5b} regions. No label remains in γ_1, γ_2 or γ_4, and expression is weakened in γ_5 and β'_2. **(E)** R15A04-GAL80 suppresses all expression in α_1 and β'_1 innervating neurons when combined with R58E02-GAL4 driven GFP and weakens labeling in β_2 and γ_5. **(F)** R15A04-GAL80 removes labeling of ~4 somata and reduces innervation in β_2 when combined with 0279-GAL4;UAS-mCD8::GFP. Scale bar, 50 µm and applies to D-F. **(G)** R58E02-GAL80 suppressed all 0804-GAL4;UAS-mCD8::GFP expression in PAM neurons. **(H)** R15A04-GAL80 removed all expression in PAM neurons in 0804-GAL4;UAS-mCD8::GFP. **(I)** *TH*-GAL80 left expression of all ~eight 0804-GAL4 labeled PAM neurons intact, showing that γ_{5n} neurons are not covered by *TH*-GAL80. Scale bar, 50 µm and applies to G-I. **(J)** R15A04-GAL80 combined with R56H09-GAL4;UAS-mCD8::GFP completely suppresses expression in γ_{5n}. The new cell type in γ_1, γ_2 and γ_4 remains, as does strong expression in β'_{2am} and weaker labeling in β'_{2p}. Projection views of 25 and 15 individual 1 µm confocal sections at the level of the γ and β/β' lobes, respectively. Scale bar 20 µm. **(K)** Expression of UAS-DenMark (magenta) and UAS-Dsyd1::GFP (green) reveals that all PAM neuron innervation of the horizontal lobes in R15A04-GAL4 flies is presynaptic. **(L)** DenMark (magenta) and Dsyd1::GFP (green) reveals that all 0804-GAL4 expressing PAM neurons are presynaptic in the horizontal lobes. **(M)** R87D06-GAL4 driven DenMark (magenta) and Dsyd1::GFP (green) reveals that all PAM neurons are presynaptic in the horizontal lobes. Scale bars in K-M 20 µm.

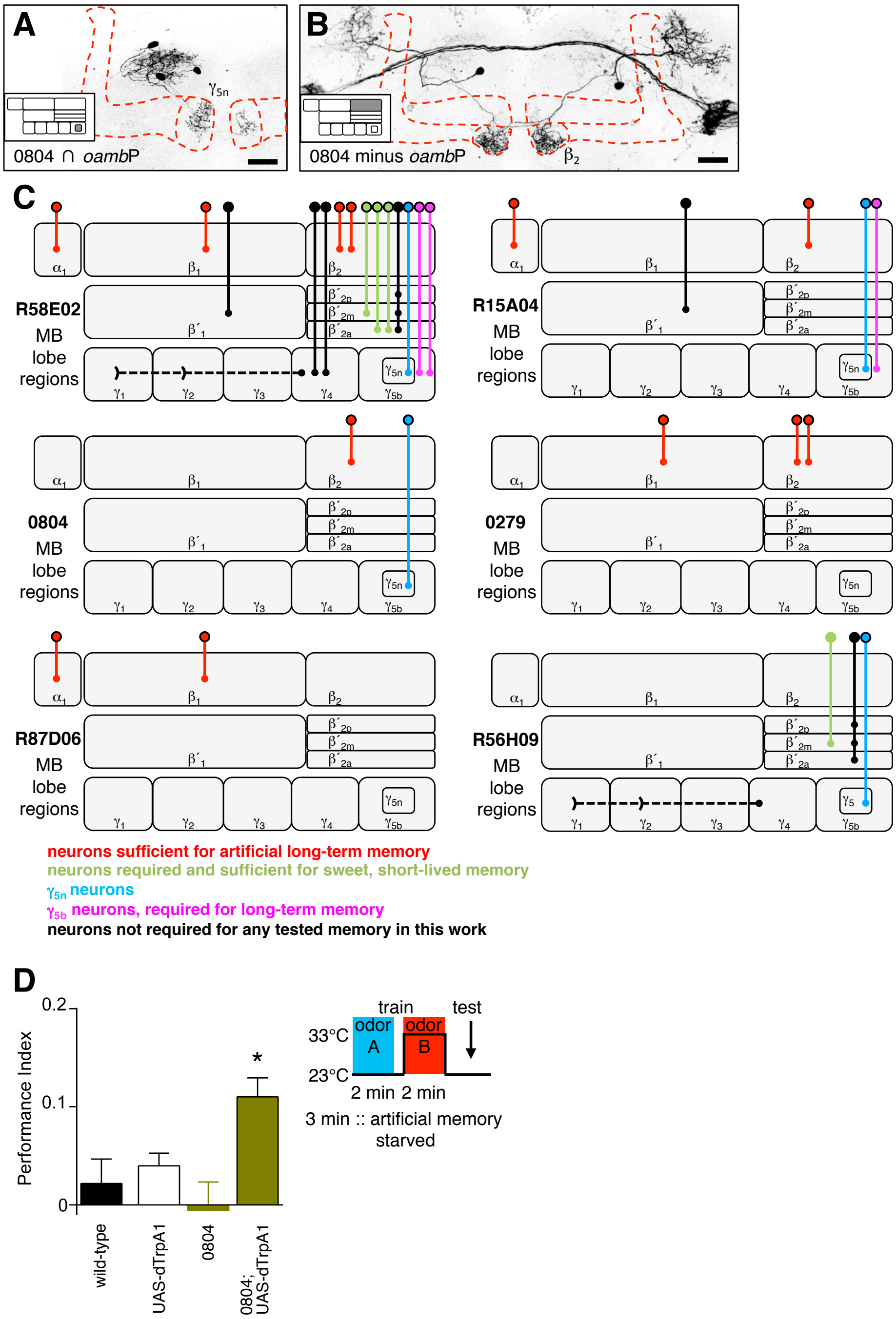

Figure S4. Anatomical detail of dopaminergic neurons innervating the horizontal mushroom body lobes. Data related to Figures 1-4.

(A) Positive genetic intersection between 0804-GAL4 and *oamb*P-LexA using lexAop-FLP in combination with UAS>STOP>GFP reveals mosaic unilateral labeling of three γ_{5n} neurons that innervate a narrow band in the ipsilateral γ_5 and form a commissure to an even smaller innervation in the contralateral γ_5. **(B)** Subtracting *oamb*P-LexA neurons from 0804;UAS-mCD8::GFP expression using lexAop-GAL80

reveals 2 cells innervating only the β_2 zones. Since *oamb*P-GAL4-driven UAS-*dTrpA1* cannot implant LTM whereas 0804;UAS-*dTrpA1* can form robust LTM, these two neurons could be those responsible for reinforcing appetitive LTM (Fig. 3A). Scale bars in A, B 20 μm. **(C)** Schematics of the horizontal mushroom body lobes and the respective regions innervated by R58E02, R15A04, 0804, 0279, R87D06, and R56H09. Red labeled cells are sufficient to implant artificial LTM, green labeled cells are required for sweet-taste reinforced STM. The blue γ_{5n} neuron type is found in all lines except 0279 and R87D06. The magenta cell type γ_{5b} is essential for appetitive LTM formation. The black neurons were apparently not critical for any of the behavioral phenomena in this study. **(D)** Artificially implanted memory using 0804-GAL4 driven UAS-*dTrpA1* can guide behavior immediately after training in starved flies ($p < 0.001$, ANOVA, $n \geq 11$), consistent with residual STM performance in Tbh^{M18} mutant flies (Fig. 1A).

dense (black) or partial (grey) coverage in respective region
sufficient for artificial long-term memory
required and sufficient for sweet, short-lived memory
required for long-term memory

	0273	R58E02	R15A04	0104	0104 minus R48B04	R48B04 =*oambP*	R48B04 minus 0104	0279	0804	R87D06	R56H09	TH*	NP1528* NP5272*
α_1	●	●	●							●			
β_1	●	●						●		● (grey)			
β_{2SC}	●	●	● (grey)	●				●	● (grey)				
β_{2s}	●											●	●
β'_1	●	●	●										
β'_{2a}	●	●		● (grey)		●	● (grey)				● (grey)		
β'_{2m}	●	● (grey)		●	● (grey)	● (grey)					● (grey)		
β'_{2p}	●	●		● (grey)		● (grey)					● (grey)		
γ_1												●	
γ_2												●	
γ_3	●	●											
γ_4	●	●		● (grey)		●	● (grey)						
γ_{5b}	●	●	● (grey)	●	●							● (grey)	
γ_{5n}	●	●	●			●	●		●		●		
MB-M9	●	●		●		●					●		

* TH-GAL4 expression according to Aso et al. 2012, modified

Supplementary Table 1. Overview of dopaminergic neurons labeled in each GAL4 line detailing their zonal innervation within the mushroom body lobes. Black dots represent coverage of the respective line in that region; grey dots represent partial coverage. Red background denotes regions which can form artificial LTM, if stimulation of their respective dopaminergic neurons is paired with odor exposure. Green background marks regions innervated by neurons that are required for sweet-taste reinforced STM. Magenta background marks the γ_{5b} cell type that is required for nutrient LTM formation. Innervation information for TH-GAL4 and the MB-M3 lines NP1528 and NP5272, that can mediate aversive reinforcement, is taken

from previous studies [17, 33, 34]. Abbreviations: α_1, β_1, β'_1, γ_1, γ_2, γ_3, γ_4, and γ_5: regions of the basal vertical α lobe, horizontal β, β', and γ lobe respectively; β_{2sc}: dopaminergic neuron type covering both core and surface in β_2; β_{2s}: surface region only in the β lobe tip, covered by dopaminergic neuron type MB-M3; β'_{2a}: anterior part of the β'lobe tip; β'_{2m}: median part of the β' lobe tip; β'_{2p}: dorsoposterior part of the β'lobe tip; γ_{5b}: broad innervation of γ_5, the distal γ lobe tip; γ_{5n}: cell type that leads to a narrow-banded innervation pattern of the γ lobe tip. MB-M9: new PAM cell-type that covers g_1, g_2, and g_4 and is not labeled with anti-TH antibody.

Additional References:

34. Aso, Y., Herb, A., Ogueta, M., Siwanowicz, I., Templier, T., Friedrich, A.B., Ito, K., Scholz, H., and Tanimoto, H. (2012). Three dopamine pathways induce aversive odor memories with different stability. PLoS Genet. 8, e1002768
