# Supplementary material for: Sweet Taste and Nutrient Value Subdivide Rewarding Dopaminergic Neurons in Drosophila
Source: Curr Biol. 2015 Mar 16;25(6):751–8. doi: 10.1016/j.cub.2015.01.036 (PMC4372253; doi:10.1016/j.cub.2015.01.036)
Supplement: Document S2. Article plus Supplemental Information [file mmc2.pdf]

Report

Current Biology

Sweet Taste and Nutrient Value Subdivide Rewarding Dopaminergic Neurons in *Drosophila*

Highlights

- Sweet taste and nutrient value recruit different reinforcing dopaminergic neurons
- Sweetness and nutrient value separately reinforce short- and long-term memories
- Reinforcement of short-term memory is not dependent on the state of hunger
- Acquisition and retrieval of long-term memory are hunger state dependent

Authors

Wolf Huetteroth, Emmanuel Perisse, ..., Christopher Burke, Scott Waddell

Correspondence

scott.waddell@cncb.ox.ac.uk

In Brief

A small number of dopaminergic neurons in the fly brain are crucial for appetitive memory reinforcement. Huetteroth et al. further subdivide these rewarding neurons into those required for sweet taste reinforcement of short-term memory, nutrient-dependent long-term memory, and others that can artificially reinforce food-independent long-term memory.

CrossMark

Huetteroth et al., 2015, Current Biology *25*, 751–758
March 16, 2015 ©2015 The Authors
http://dx.doi.org/10.1016/j.cub.2015.01.036

CellPress

Current Biology *25*, 751–758, March 16, 2015 ©2015 The Authors http://dx.doi.org/10.1016/j.cub.2015.01.036

Report

Sweet Taste and Nutrient Value Subdivide Rewarding Dopaminergic Neurons in *Drosophila*

Wolf Huetteroth,[1,2] Emmanuel Perisse,[1] Suewei Lin,[1] Martín Klappenbach,[1,3] Christopher Burke,[1] and Scott Waddell[1,*]
[1]Centre for Neural Circuits and Behaviour, The University of Oxford, Tinsley Building, Mansfield Road, Oxford OX1 3SR, UK
[2]Zukunftskolleg, University of Konstanz, Universitätsstraße 10, 78457 Konstanz, Germany
[3]Laboratorio de Neurobiología de la Memoria, Departamento de Fisiología y Biología Molecular y Celular, IFIBYNE-CONICET, Pabellón II, Facultad de Ciencias Exactas y Naturales, Universidad de Buenos Aires, Buenos Aires C1428EGA, Argentina

Summary

Dopaminergic neurons provide reward learning signals in mammals and insects [1–4]. Recent work in *Drosophila* has demonstrated that water-reinforcing dopaminergic neurons are different to those for nutritious sugars [5]. Here, we tested whether the sweet taste and nutrient properties of sugar reinforcement further subdivide the fly reward system. We found that dopaminergic neurons expressing the OAMB octopamine receptor [6] specifically convey the short-term reinforcing effects of sweet taste [4]. These dopaminergic neurons project to the β'_2 and γ_4 regions of the mushroom body lobes. In contrast, nutrient-dependent long-term memory requires different dopaminergic neurons that project to the γ_{5b} regions, and it can be artificially reinforced by those projecting to the β lobe and adjacent α_1 region. Surprisingly, whereas artificial implantation and expression of short-term memory occur in satiated flies, formation and expression of artificial long-term memory require flies to be hungry. These studies suggest that short-term and long-term sugar memories have different physiological constraints. They also demonstrate further functional heterogeneity within the rewarding dopaminergic neuron population.

Results and Discussion

Sweet taste and nutrient value of sugars reinforce learning in *Drosophila* [7, 8]. Octopaminergic neurons specifically convey sweet taste signals [4, 9]. Blocking them impaired short-term memory (STM) reinforced by the sweet but non-nutritious arabinose. In contrast, long-term memory (LTM) formed with sweet and nutritious sucrose was unaffected. Reinforcing octopamine activates a subpopulation of dopaminergic neurons via the Ca^{2+}-coupled α-adrenergic-like octopamine receptor OAMB. However, despite the evident separation of memory phases with octopamine [4, 10], manipulating dopaminergic neurons has so far impacted sweet taste and nutrient-reinforced memory [3, 4]. We therefore investigated whether octopamine dependence separates rewarding dopaminergic neurons.

*Correspondence: scott.waddell@cncb.ox.ac.uk
This is an open access article under the CC BY license (http://creativecommons.org/licenses/by/4.0/).

CrossMark

Tbh^{M18} mutant flies, lacking octopamine, cannot form STM reinforced with 2 M sucrose [11]. However, a persistent memory slowly emerges after training *Tbh*^{M18} flies with odor and 1 M sucrose [10], suggesting that nutrient-dependent LTM is likely to be formed in parallel and independent of appetitive STM. Since nutrient-dependent memory can guide behavior as quickly as 2 min after training [7], we first determined whether nutrient memory could be observed in wild-type and *Tbh*^{M18} flies trained with saturated sucrose, ~5.8 M (Figure 1A). Strikingly, this analysis revealed performance at all times in *Tbh*^{M18} flies that was statistically different to wild-type immediately after training but indistinguishable from wild-type 30 min, 3 hr, and 24 hr after training. These data are consistent with *Tbh*^{M18} flies only lacking sweet-taste-reinforced STM [4, 10]. Moreover, they demonstrate that nutrient-dependent (octopamine-independent) memory is observable immediately after training with high sucrose concentrations.

Prior knowledge that octopamine activates rewarding dopaminergic neurons through the OAMB receptor [4] led us to identify R48B04-GAL4 in the FlyLight collection [13]. R48B04-GAL4 is driven by a promoter fragment from the *oamb* gene (although we acknowledge that this reagent is unlikely to label all *oamb*-expressing neurons, from here on, we will refer to it as *oamb*P-GAL4). We verified the relevance of *oamb*P-GAL4 neurons by knocking down OAMB expression with UAS-*oamb*^{RNAi} [4, 14]. As expected, these flies completely lacked STM when trained with the sweet and non-nutritious sugar arabinose (Figure 1B). The memory defect was more pronounced than when OAMB was knocked down in dopaminergic neurons with 0104-GAL4 [4], suggesting *oamb*P-GAL4 may more accurately label octopamine-responsive dopaminergic neurons than 0104-GAL4 (Figures S1A–S1E). Our initial examination of *oamb*P-GAL4 revealed expression in approximately 55 rewarding dopaminergic neurons (and ~12 tyrosine hydroxylase (TH)-negative neurons) in the protocerebral anterior medial (PAM) cell cluster that innervate the horizontal mushroom body lobes (Figures 1C–1E, S1A, S1C, and S1D; [5]).

We next tested the contribution of *oambP*-GAL4 neurons to saturated sucrose-reinforced memory by blocking their output using the dominant temperature-sensitive UAS-*shibire*^{ts1} (UAS-*shi*^{ts1}) transgene [15]. Blocking *oamb*P-GAL4 neurons significantly impaired STM (Figure 1F). However, LTM performance of *oamb*P-GAL4;UAS-*shi*^{ts1} flies was indistinguishable from controls, demonstrating a specific loss of STM (Figure 1G), consistent with *Tbh*^{M18} flies trained with sucrose (Figure 1A).

We also tested a reinforcing role of *oambP*-GAL4 neurons by pairing their activation, using UAS-*dTrpA1*, with odor presentation (Figures 1H and 1I). The *dTrpA1*-encoded transient receptor potential (TRP) channel conducts Ca^{2+} and depolarizes neurons when temperature exceeds 25°C [16]. This protocol implanted STM that was statistically different from all controls in both starved and fed flies (Figures 1H and S1F). However, implanted memory did not persist. Performance of *oamb*P-GAL4;UAS-*dTrpA1* flies was indistinguishable from controls 24 hr after training (Figure 1I). Taken together, these

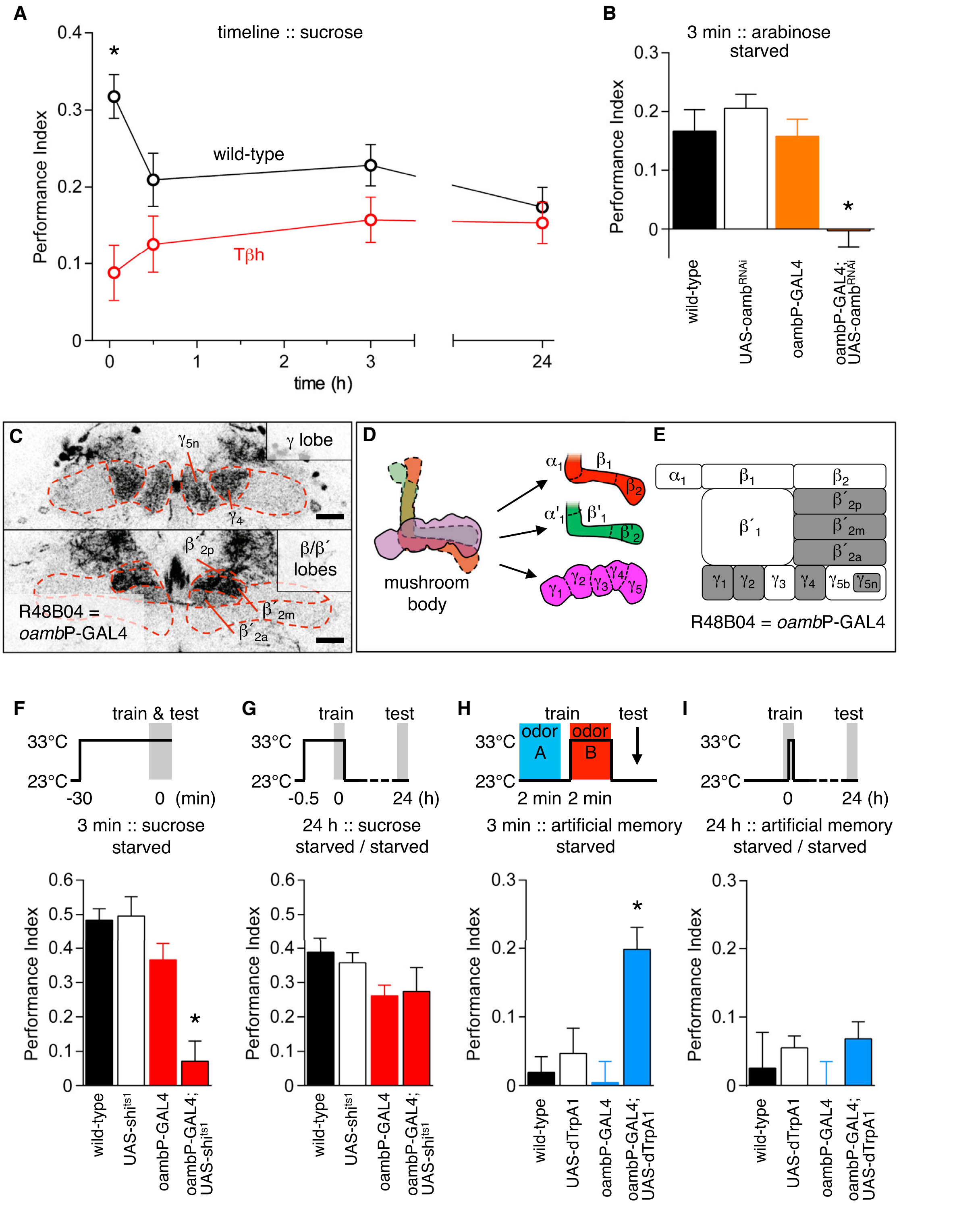

Figure 1. Sweet Taste Reinforces Short-Term Memory via Octopamine Signaling in *oamb*P-GAL4 Dopaminergic Neurons

(A) Tbh^{M18} flies exhibit defective STM following training with concentrated sucrose (compared to wild-type, $p < 0.0001$, t test). Residual memory of Tbh^{M18} flies persists and is statistically indistinguishable from memory in wild-type flies 30 min, 3 hr, and 24 hr after training (all $p > 0.7$, t test). All $n \geq 12$.

(B) Hungry *oamb*P-GAL4;UAS-*oamb*^{RNAi} flies lack STM following training with arabinose (versus controls, $p < 0.0001$, ANOVA, $n \geq 8$).

(C) R48B04 (referred to as *oamb*P-GAL4) labels about 55 dopaminergic neurons that zonally innervate γ_1, γ_2, γ_4, and γ_{5n} of the γ lobe and β'_{2a}, β'_{2m}, and β'_{2p} of the β' lobe. 1.5-µm frontal confocal sections at the level of the γ lobe and β lobes are shown; scale bars represent 20 µm. See Figure S1A for full brain expression.

(D) Schematic of the mushroom body lobes and additional zonal suborganization of the horizontal β (red), β' (green), and γ (magenta) lobes. The β_1, β_2 and β'_1, β'_2 border their respective α_1 and α'_1 subregions on the base of the vertical lobes. The exclusively horizontal γ lobe can be split into γ_1–γ_5 [12].

(E) Illustration of the lobe subregions, highlighting those innervated by *oamb*P-GAL4 dopaminergic neurons (gray).

(legend continued on next page)

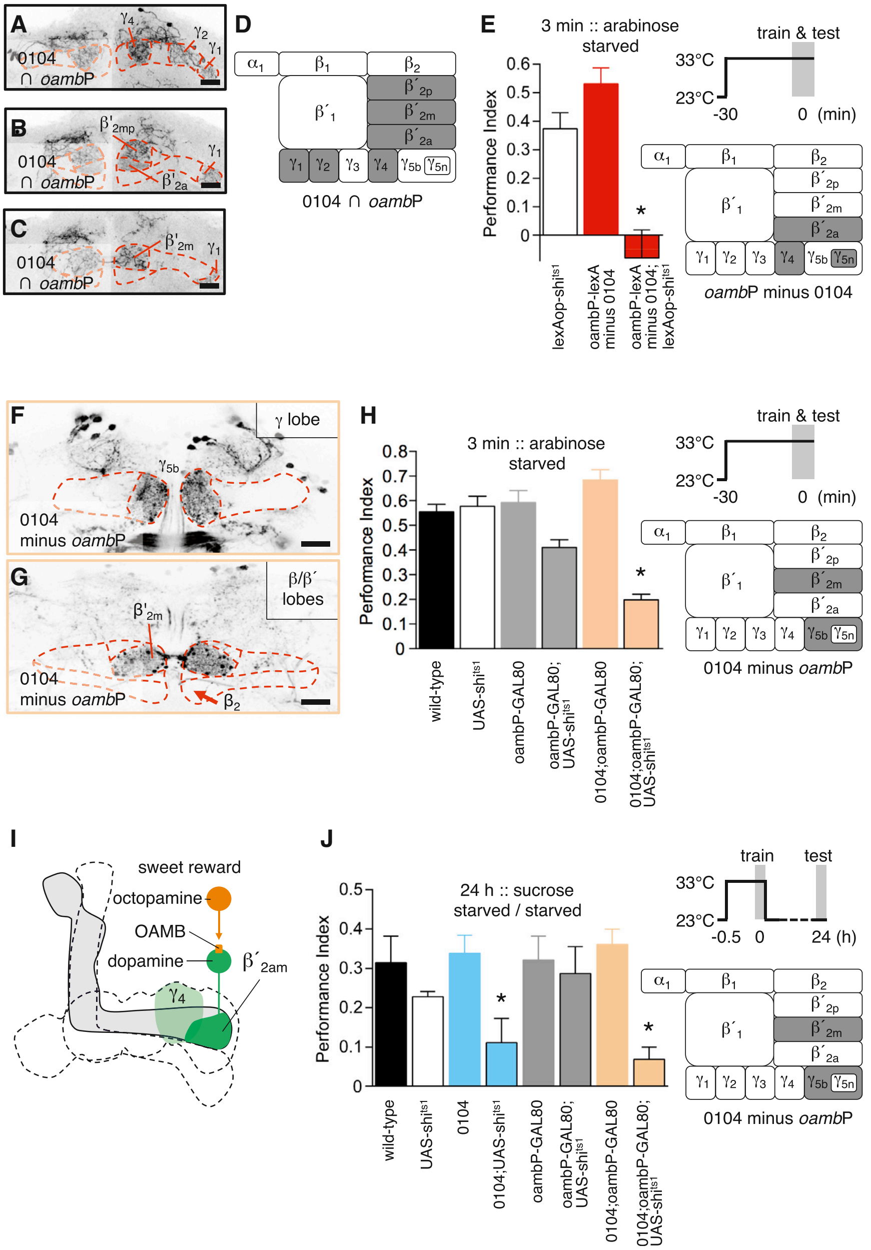

Figure 2. Short-Term Memory Reinforcement by Sweet Taste Requires β'_2 and γ_4 Dopaminergic Neurons

(A–C) Positive intersection between 0104-GAL4 and *oamb*P-lexA with lexAop-FLP,tub>GAL80> STOP and UAS-mCD8::GFP labels about 20 neurons.

(A) Projection of 20 1-μm confocal sections at the level of the γ lobe reveals processes in γ_1, γ_2, and γ_4.

(B) Projection of ten 1-μm confocal sections at the level of the β and β' lobes shows innervation in β'_{2a} and β'_{2mp} and some posterior γ_1 from the same cell type as in (A).

(C) Projection of ten 1-μm confocal sections of the β' lobe reveals shared 0104-GAL4 and *oamb*P-LexA innervation of β'_{2m}.

Scale bars of (A)–(C) represent 20 μm.

(D) Illustration summarizing the zones of the mushroom body innervated by neurons common to 0104-GAL4 and *oamb*P-lexA.

(E) *oamb*P neurons not labeled by 0104 are required for STM formation with arabinose. Performance of starved *oamb*P-LexA/lexAop-*shi*^{ts1}; 0104-GAL4/UAS-*lexA*^{RNAi} flies is significantly different to controls ($p < 0.0001$, ANOVA, n = 8). See permissive temperature control in Figure S1G. Illustration demonstrates dopaminergic neurons unique to *oamb*P-lexA/*oamb*P-GAL4.

(F and G) Removing *oamb*P neurons from 0104 (*oamb*P-GAL80/20xUAS-6xGFP;0104-GAL4) leaves expression in about 15 neurons innervating γ_{5b} (F) and β'_{2m} (G) neurons. Projection of five 2-μm confocal sections covering the γ lobe (F) and the β/β' lobe (G). *oamb*P-GAL80 also unexpectedly suppresses expression in the β_2 (red arrow), which is otherwise labeled by 0104-GAL4 (Figure S1B). Scale bars represent 20 μm.

(H) Blocking output from γ_{5b} and β'_{2m} dopaminergic neurons, shown in illustration, during arabinose-reinforced training impairs STM. Performance of *oamb*P-GAL80;0104-GAL4/UAS-*shi*^{ts1} flies was significantly different to controls ($p < 0.0001$, ANOVA, n = 8). See permissive temperature control in Figure S1H.

(I) Summary of STM formation. Sweet taste engages octopaminergic neurons. Octopamine acts through the OAMB receptor to activate rewarding dopaminergic neurons that innervate β'_{2am} (and γ_4) regions of the mushroom body.

(J) Blocking output from the β'_{2m} and γ_{5b} subset of 0104-GAL4 neurons during sucrose-reinforced training impairs 24-hr LTM. Performance of 0104-GAL4/UAS-*shi*^{ts1} and *oamb*P-GAL80;0104-GAL4/UAS-*shi*^{ts1} flies was significantly different to controls ($p < 0.0002$, ANOVA, n = 5-12). See permissive temperature control in Figure S2B.

data suggest that *oamb*P-GAL4 dopaminergic neurons specifically convey octopamine-dependent and hunger-state-independent sweet taste reinforcement, whereas other rewarding dopaminergic neurons contribute nutrient value signals.

0104-GAL4 labels octopamine-responsive dopaminergic neurons and some required for nutrient reinforcement [4]. We reasoned that intersecting 0104-GAL4 and *oamb*P-GAL4 would separate sweet and nutrient reinforcement. Common neurons in 0104-GAL4 and *oamb*P-GAL4 can be visualized by combining R48B04-LexA (i.e., *oamb*P-LexA, which expresses in *oamb*P-GAL4 dopaminergic neurons; Figure S1C) with 0104-GAL4-driven UAS>STOP>GFP (where > represents a FLP-recombinase target sequence) and lexAop-*FLP*. In these flies, GFP labels 10–20 dopaminergic neurons innervating the anterior, median, and posterior β'_2 (β'_{2amp}) and γ_4 zones of the mushroom body, in addition to a new class of TH-negative neurons that connect γ_1, γ_2, and γ_4 (Figures 2A–2D).

(F and G) Blocking *oamb*P-GAL4 neuron output with UAS-*shi*^{ts1} significantly impairs STM in starved flies trained with sucrose ($p < 0.0001$, ANOVA, n = 8) (F), whereas it has no effect on 24-hr LTM, as compared to controls ($p > 0.1$, ANOVA, n = 8) (G).

(H) Pairing odor exposure with dTrpA1 activation of *oamb*P-GAL4 neurons forms STM that is significantly different to control flies ($p < 0.0002$, ANOVA, $n \geq 9$).

(I) Artificially implanted *oamb*P-GAL4 memory is labile. Performance of hungry *oamb*P-GAL4; UAS-*dTrpA1* flies is not statistically different to that of controls at 24 hr ($p > 0.1$, ANOVA, $n \geq 10$).

Both *oamb*P-GAL4 and 0104-GAL4 contain γ_5-innervating neurons (Figures 1C and S1A–S1E), but the positive intersection does not label them, suggesting that each GAL4 includes unique γ_5 neurons: $\gamma_{5narrow}$ (γ_{5n}) in *oamb*P-GAL4 (Figures 1C, S1A, S1C, and S1D) and γ_{5broad} (γ_{5b}) in 0104-GAL4 (Figures S1B and S1E).

To assess the role of subsets of *oamb*P-labeled neurons, we removed expression in 0104 neurons by combining 0104-GAL4 with UAS-*lexA*^{RNAi},*oamb*P-LexA flies and a lexAop-*shi*^{ts1} transgene, thereby restricting expression to dopaminergic neurons innervating β'_{2a}, γ_4, and γ_{5n} [5]. These flies exhibited no STM following training with sweet-only arabinose at restrictive 33°C (Figure 2E). No significant defect was evident at permissive 23°C (Figure S1G). We also constructed 0104-GAL4;*oambP*-GAL80 flies in which GAL80 inhibits GAL4-driven gene expression [17] resulting in expression being restricted to β'_{2m} and γ_{5b} 0104 neurons (Figures 2F–2H). These flies also displayed defective STM following conditioning at restrictive 33°C with arabinose (Figure 2H), while no significant defect was evident at permissive 23°C (Figure S1H). Since blocking γ_{5b} and γ_{5n} neurons with R15A04-GAL4 does not impair STM (Figure S2A), we conclude that sweet taste reinforcement is conveyed by octopaminergic signaling through the OAMB receptor in dopaminergic neurons that innervate the β'_{2am} and γ_4 zones of the mushroom body (Figure 2I).

0104-GAL4 also includes neurons required for nutrient-dependent LTM, which are not in *oamb*P-GAL4. Indeed, blocking 0104-GAL4;*oambP*-GAL80 neurons with UAS-*shi*^{ts1} revealed a significant LTM defect (Figure 2J). No defects were apparent at the permissive temperature (Figure S2B). These data indicate that dopaminergic neurons in β'_{2m} and/or γ_{5b} are required for nutrient-dependent LTM formation.

We next visually screened for GAL4 lines with expression in PAM dopaminergic neurons that innervate the horizontal mushroom body lobes. We used these and three established PAM lines [3, 4, 18] to express UAS-*shi*^{ts1} and tested LTM following sucrose-reinforced learning. Blocking 0273, R58E02, or R15A04 neurons during training significantly impaired LTM performance compared to the relevant controls (Figure 3A). In contrast, blocking 0279, 0804, R87D06, or R56H09 neurons did not. No significant defects were apparent when R15A04;UAS-*shi*^{ts1}, R58E02;UAS-*shi*^{ts1}, or 0273;UAS-*shi*^{ts1} flies were trained and tested at permissive 23°C (Figure S2C).

0273 and R58E02 label ~130 and ~90 dopaminergic neurons, respectively, that broadly innervate the horizontal lobes (Figures 3B and 3C; [3, 4]). R15A04 expresses in ~26 dopaminergic neurons projecting to α_1, β'_1, β_2, γ_{5b}, and γ_{5n} (Figures 3D, S2D, and S3K). These overlap with 0104 in β_2 and γ_{5b}. Ineffective GAL4 lines further refine necessary nutrient-reinforcing neurons (Figures S3 and S4A–S4C; Table S1). Briefly, 0279-GAL4 dopaminergic neurons innervate β_1 and β_2 (Figure 3E; [18]). 0804-GAL4 innervate β_2 and γ_{5n} (Figures 3F, S2E, and S3L), R87D06-GAL4 project to α_1 and β_1 (Figures 3G, S2F, and S3M), and R56H09-GAL4 innervate β'_{2m} and γ_{5n} (Figures 3H, S2G, and S3J). These negative data indicate that β_2 and γ_{5n} innervation is dispensable (Figures 3A, 3E–3H, and S4C). Therefore, we conclude that nutrient reinforcement requires dopaminergic neurons innervating γ_{5b} (Figure 3I).

Artificially activating large groups of 0273 or R58E02 dopaminergic neurons paired with odor formed robust appetitive memory [3, 4]. We therefore tested for subsets that were sufficient to reinforce LTM (Figure 3J). We combined each GAL4 with UAS-*dTrpA1* and paired dTrpA1-activating 33°C with odor. Surprisingly, 0273, R58E02, 0104, 0279, R15A04, 0804, and R87D06 produced LTM performance that was statistically different to their relevant control flies, whereas R56H09 did not (Figure 3J). Notably, 0279, 0804, and R87D06 neurons, which were not required for sucrose LTM, reinforced artificial 24-hr memory. These and all other LTM-competent lines (0273, R58E02, 0104, R15A04) include dopaminergic innervation of the β lobe or adjacent α_1 lobe (Figures 3B–3G, S1B, S1E, and S4C; Table S1), whereas those that cannot implant LTM lack projections to these regions (R48B04, R56H09; Figures 1C–1E, 3H, S1A, S1C, S1D, S3J, and S4C; Table S1). Therefore, we conclude that artificial LTM can be formed by dopaminergic neurons innervating α_1, β_1, and β_2 (Figure 3K). Furthermore, removal of STM-reinforcing *oamb*P-LexA neurons (Figures 1H and 1I) from 0804 (Figure S4A) leaves expression in only two neurons innervating β_2 (Figure S4B), suggesting that these alone may provide sufficient reinforcement for appetitive LTM. Such localization of lasting reinforcement is consistent with the importance of $\alpha\beta$ neurons for LTM and its retrieval [20–23].

Given the discordance between the γ_{5b} neurons required for sucrose LTM and those targeting α_1, β_1, and β_2 that can reinforce persistent memory, we tested the food relevance of implanted memories. Expression of sugar-reinforced memory, but not water-reinforced memory, can be suppressed by feeding flies after training [5]. Feeding suppressed LTM performance of 0104;UAS-*dTrpA1*, R15A04; UAS-*dTrpA1*, 0804;UAS-*dTrpA1*, and R87D06;UAS-*dTrpA1* flies to levels that were statistically indistinguishable from their respective fed controls (Figure 4A). In contrast, significant performance remained in 0279;UAS-*dTrpA1* and 0273;UAS-*dTrpA1* flies. Therefore, memory reinforced in the β and adjacent α_1 regions by 0104, R15A04, 0804, and R87D06 neural activation mimics sucrose-reinforced memory, whereas 0279-implanted memory has different properties.

Although flies ordinarily need to be hungry to form sugar-reinforced appetitive memory, prior experiments and those here demonstrate that appetitive STM can be formed in fed flies by pairing octopaminergic or dopaminergic neuron activation with odor presentation (Figure S1F; [3, 4]). We therefore tested whether nutrient-dependent LTM could also be formed artificially in food-satiated flies. We analyzed food-relevant 0104-formed and R87D06-formed memory and non-food satiable 0279-formed and 0273-formed memory in parallel. Strikingly, 0104, R87D06, and 0279 activation did not form LTM in satiated flies, whereas 0273;UAS-*dTrpA1* flies exhibited robust LTM (Figure 4B), which was even evident following 7 days of ad libitum feeding after training (Figure 4C). Satiety therefore also constrains the artificial formation of appetitive LTM. We speculate that some 0273 dopaminergic neurons represent rewarding events other than food.

Taken with prior studies [4, 7, 10], results here demonstrate that the sweet taste and nutrient properties of sugars are independently processed and reinforce memories of different duration. Sweet taste is transduced through octopaminergic neurons whose released octopamine, via the OAMB receptor, activates dopaminergic neurons that project to the β'_{2am} and γ_4 regions of the mushroom body. Octopaminergic reinforcement also modulates the state dependence of STM via the

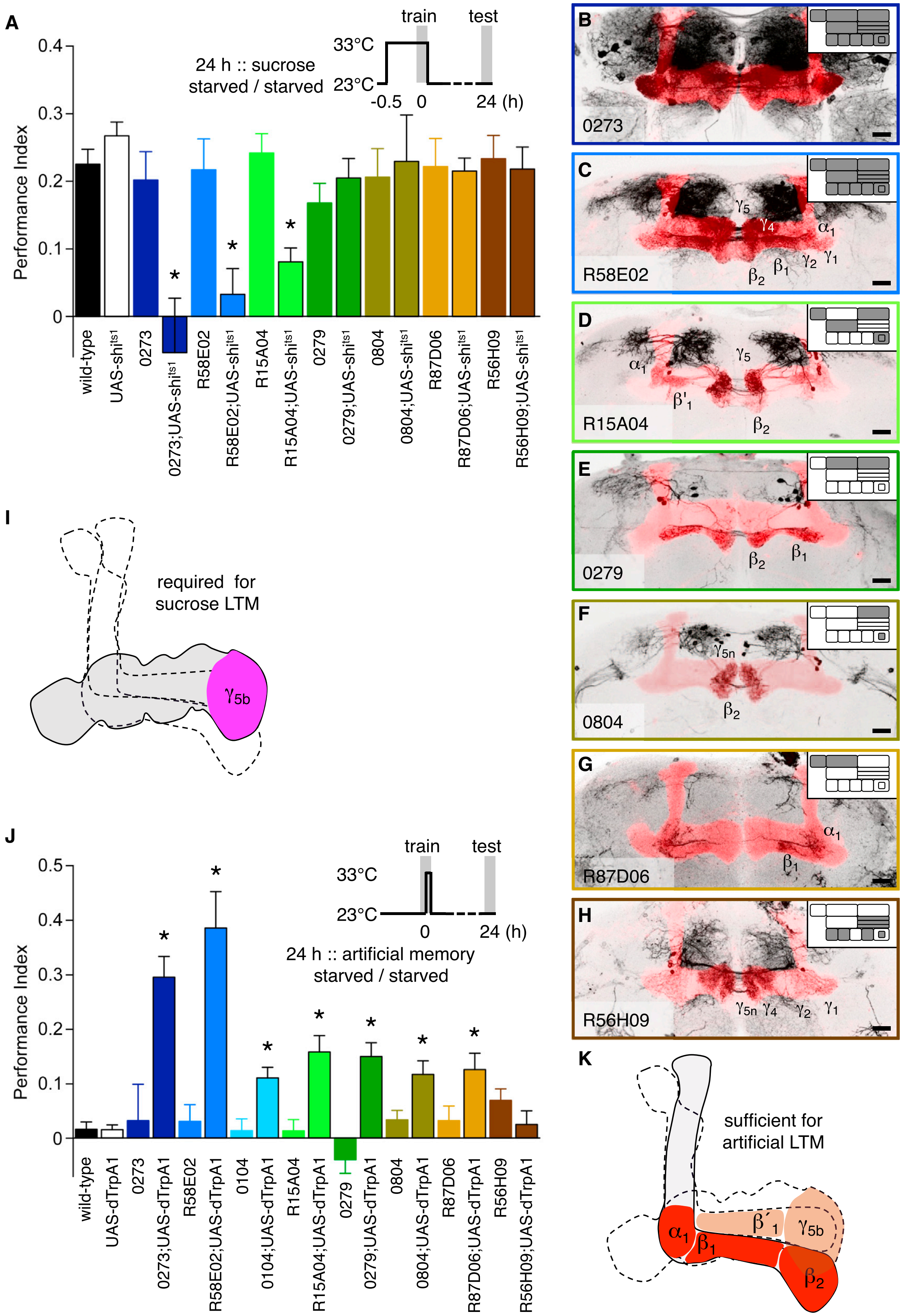

Figure 3. Dopaminergic Neurons Required for Nutrient-Dependent LTM Differ from Those that Can Artificially Implant LTM

(A) Blocking output from 0273, R58E02, and R15A04 neurons during sucrose training significantly disrupted LTM in starved flies (all $p < 0.001$, ANOVA, $n \geq 9$). See permissive temperature control in Figure S2C. LTM was not statistically impaired by blocking 0279, 0804, R87D06, or R56H09 neurons ($p > 0.7$, ANOVA, $n \geq 8$).

(legend continued on next page)

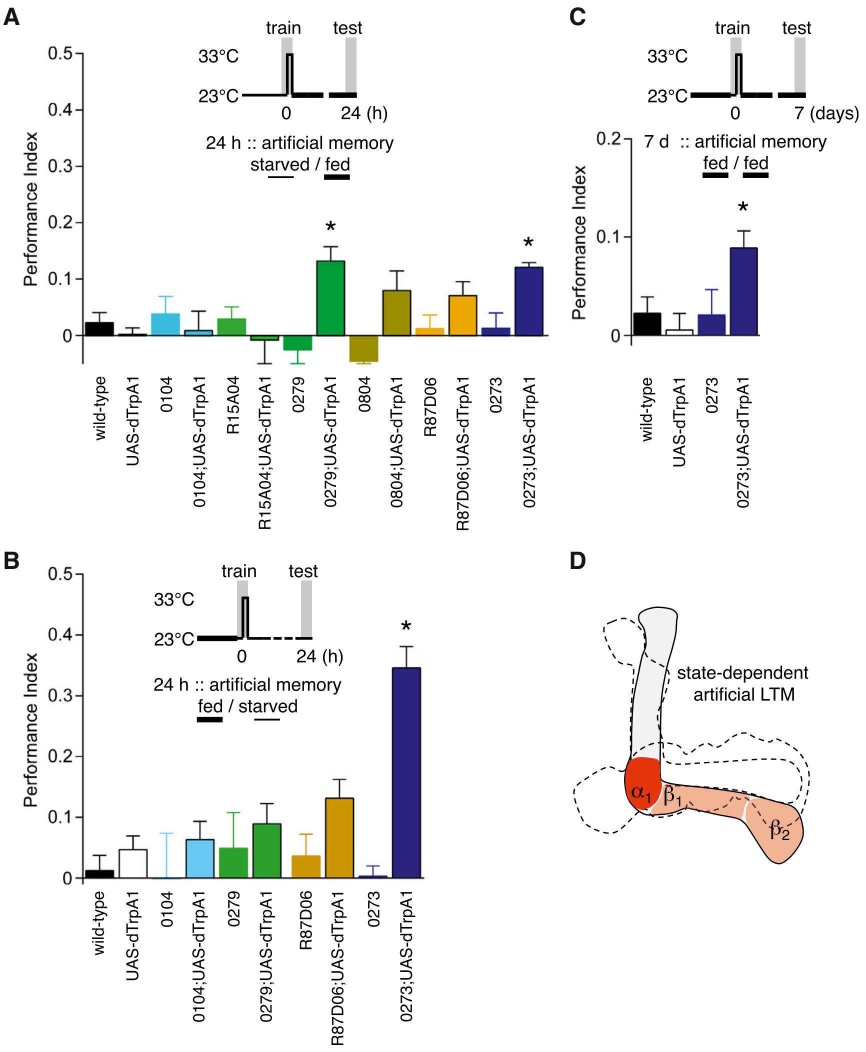

Figure 4. Distinct Dopaminergic Neurons Form Hunger-State-Dependent and Hunger-Independent LTM

(A) Feeding after training suppresses artificially implanted LTM performance in 0104, R15A04, 0804, and R87D06 flies expressing UAS-*dTrpA1* (all $p > 0.1$, ANOVA, $n \geq 9$). Statistically significant memory remained after feeding in 0279;UAS-*dTrpA1* and 0273;UAS-*dTrpA1* flies ($p < 0.0001$, ANOVA, $n \geq 8$). No other group showed performance that was statistically different to their respective fed controls ($p > 0.08$, ANOVA, $n \geq 9$).

(B) Significant LTM could not be formed when odor was paired with UAS-*dTrpA1*-driven activation of 0104, 0279, or R87D06 neurons in food-satiated flies, despite 24 hr of food deprivation after training ($p > 0.2$, ANOVA, $n \geq 6$). In contrast, significant memory was formed in food-satiated 0273;UAS-*dTrpA1* flies ($p < 0.0001$, ANOVA, $n \geq 6$).

(C) Measurable artificial LTM remained in 0273;UAS-*dTrpA1* flies following 7 days of ad libitum feeding after training ($p = 0.001$, ANOVA, $n \geq 6$).

(D) Summary. Formation and retrieval of LTM by dopaminergic neurons innervating α_1, β_1, and β_2 are sensitive to feeding before and/or after training.

OCTβ2R receptor that is required in the dopaminergic MB-MP1 neurons [4].

Nutrient-dependent LTM does not involve octopamine [4, 10] or sweet-taste-reinforcing dopaminergic neurons. Nutrient reinforcement instead requires dopaminergic neurons innervating γ_{5b} of the mushroom body, whereas those going to β_1, β_2, and the adjacent α_1 region are sufficient. More work will be required to understand this distributed process, which apparently has an immediate and delayed dynamic (Figure S4D; [7, 10]).

Whereas formation and expression of sweet-taste-reinforced STM is insensitive to satiety state, artificial formation and expression of nutrient-relevant memory require flies to be hungry. Even direct stimulation of the relevant rewarding dopaminergic neurons cannot implant appetitive LTM in food-satiated flies. These experiments suggest that hunger establishes an internal state that permits the nutrient-reinforcing signals to be effective. It will be interesting to understand what the permissive state involves and where it is required. Others have previously described a role for CRTC in enabling hunger-dependent LTM in the fly [24] and promoting persistent memory in the mouse [25]. It therefore seems plausible that such a mechanism might be required in the mushroom body neurons to permit nutrient-dependent reinforcement.

Experimental Procedures

Fly Strains

Fly stocks were raised on standard cornmeal food at 25°C and 50%–60% relative humidity. The wild-type *Drosophila* strain used in this study is Canton-S. The *Tbh*^{M18} mutant is described [26]. The UAS-mCD8::GFP, the 20xUAS-6xGFP, and the 247-lexA,lexAop-RFP flies are described [17, 19, 27]. The UAS-*oamb*^{RNAi} (strain number 2861GD) was obtained from the Vienna Drosophila Resource Center (VDRC) [14]. The UAS-*shi*^{ts1}, on the first and third chromosome, and UAS-*dTrpA1* transgenic strains are described [15, 16]. The R48B04, R15A04, R87D06, and R56H09 flies [13] were obtained from Bloomington. The R58E02-LexA, R58E02-GAL80, 0104, 0273, and 0279 flies are described [3, 4, 18]. The 0804 fly strain, more correctly named PBac(IT.GAL4)0804, was generated and initially characterized by Marion Sillies and Daryl Gohl as part of the InSITE collection [28]. The

(B–H) Mushroom body lobe (red) innervation of 0273-GAL4 (B) [4], R58E02-GAL4 (C) [3], R15A04-GAL4 (D), 0279-GAL4 (E) [18], 0804-GAL4 (F), R87D06-GAL4 (G), and R56H09-GAL4 (H) revealed with UAS-mCD8::GFP. The mushroom body (red) is labeled in each brain with 247-lexA::VP16-driven lexAop-rCD2::mRFP [19]. Scale bars represent 20 μm. Zonal innervation of each line is shown in the corresponding inset illustration.

(I) Summary. Dopaminergic neurons innervating γ_{5b} are essential to reinforce nutrient-dependent LTM.

(J) Pairing odor with dTrpA1 activation of 0273, R58E02, 0104, 0279, R15A04, 0804, and R87D06 dopaminergic neurons forms significant LTM (all $p < 0.001$, ANOVA, $n \geq 6$). No significant memory was formed in R56H09;UAS-*dTrpA1* flies ($p > 0.3$, ANOVA, $n \geq 10$). All flies were food deprived before and after training.

(K) Summary. Dopaminergic neurons innervating α_1, β_1, or β_2 (and perhaps β'_1 and γ_{5b}) are sufficient to form LTM.

R48B04-LexA, R15A04-GAL80, and UAS-*lexA*^{RNAi} flies are described [5]. The R48B04-GAL80 construct was made by inserting the enhancer fragment of R48B04-GAL4 from the Janelia Farm Research Campus FlyLight database [13] into the pBPGAL80Uw-6 vector (Addgene plasmid 26236). The R48B04-GAL80 fly strain was made commercially (BestGene) by site-specific insertion into the attP40 landing site. The UAS>STOP>GFP, lexAop-FLP, lexAop-GAL80, and *TH*-GAL80 strains are those employed in [29–31]. UAS-DenMark and UAS-DSyd1::GFP are those in [32] and [33].

To generate R48B04;UAS-*oamb*^{RNAi} flies, we crossed homozygous UAS-*oamb*^{RNAi} males to homozygous R48B04 females. R48B04/+ control flies were generated by crossing R48B04 females to wild-type males. Heterozygote UAS-*oamb*^{RNAi}/+ controls were generated by crossing UAS-*oamb*^{RNAi} males to wild-type females. We generated flies expressing *shi*^{ts1} in subsets of dopaminergic neurons by crossing UAS-*shi*^{ts1} females to homozygous R48B04, 0104, R48B04-GAL80;0104, R48B04-LexA;0104, 0273, R58E02, R15A04, 0279, R87D06, or R56H09 males. 0804 resides on the X chromosome; therefore, 0804 females were crossed to UAS-*shi*^{ts1} males. Heterozygote UAS-*shi*^{ts1}/+ controls were generated by crossing UAS-*shi*^{ts1} females to wild-type males. Heterozygote GAL4/+ controls were generated by crossing GAL4 males to wild-type females. We generated flies expressing *dTrpA1* in R48B04, 0273, R58E02, 0104, 0279, R15A04, R87D06, or R56H09 neurons by crossing UAS-*dTrpA1* females to homozygous R48B04, 0273, R58E02, 0104, 0279, R15A04, R87D06, or R56H09 males. Homozygous 0804 females were crossed to UAS-*dTrpA1* males. Heterozygote UAS-*dTrpA1*/+ controls were generated by crossing UAS-*dTrpA1* females to wild-type males; heterozygote GAL4/+ controls were generated by crossing GAL4 males to wild-type females and vice versa for both controls in case of 0804.

Behavior Experiments

Appetitive memory was assayed as described [22] with the following modifications. Mixed sex populations of 4- to 8-day-old flies raised at 25°C were tested together in all behavior experiments. Before training, groups of ~100 flies were food deprived for 18–22 hr in a 25-ml vial containing 1% agar and a 20 × 60 mm piece of filter paper. Training was performed with either saturated sucrose or 3 M arabinose as unconditioned stimulus. The odors used were 3-octanol (Sigma) and 4-methylcyclohexanol (Sigma) at 1:1,000 in mineral oil. Artificial memory implantation experiments using UAS-*dTrpA1*-mediated neural activation were performed as described [4]. Briefly, 8- to 11-day-old flies raised at 20°C were either kept in food vials or starved for 18–22 hr before training. Flies were presented with one odor at the permissive 23°C for 2 min in filter paper-lined tubes and were then transferred into a prewarmed filter paper-lined tube and immediately presented with a second odor at dTrpA1-channel activating 33°C for 2 min. Flies were then returned to 23°C and tested for immediate memory. To assay 24-hr memory, we transferred trained flies into either food vials or food deprivation vials until testing. For 7-day memory experiments, fed flies were trained and immediately transferred into food vials until memory testing after 7 days. Memory performance was assayed by allowing the flies 2 min to choose between the odors presented during training. Performance index (PI) was calculated as the number of flies approaching (appetitive memory) the conditioned odor minus the number of flies going to the unconditioned odor divided by the total number of flies in the experiment. A single PI value is the average score from flies of the identical genotype tested with the reciprocal combination of conditioned and unconditioned odor. Statistical analyses were performed using PRISM (GraphPad Software). Overall ANOVA was followed by planned pairwise comparisons between the relevant groups with a Tukey honestly significant difference (HSD) post hoc test. All experiments are $n \geq 8$ unless stated otherwise.

Imaging

To visualize native GFP or mRFP, we collected adult flies 2–11 days after eclosion, and brains were dissected in ice-cold 4% paraformaldehyde solution in PBS (1.86 mM NaH_2PO_4, 8.41 mM Na_2HPO_4, and 175 mM NaCl) and fixed for an additional 60 min at room temperature [19]. Samples were then washed 3 × 10 min with PBS containing 0.1% Triton X-100 (PBT) and 2 × 10 min in PBS before mounting in Vectashield (Vector Labs). Imaging of frontal brain views was performed on a Leica TCS SP5 X and a Zeiss LSM 510. The resolution of the image stacks were 1024 × 1024 with 0.5–1.5-μm step size and a frame average of 4. Images were processed in AMIRA 5.3 (Mercury Systems) and Fiji. The immunostaining against TH and GFP was performed as previously described [4].

Supplemental Information

Supplemental Information includes four figures and one table and can be found with this article online at http://dx.doi.org/10.1016/j.cub.2015.01.036.

Acknowledgments

We thank Y. Huang and R. Brain for technical support and the Bloomington Stock Center, VDRC, T. Clandinin, D. Gohl, M. Sillies, G. Rubin, and the Janelia Farm project for flies. We thank members of the S.W. and G. Miesenböck laboratories for discussion. W.H. is supported by a ZIF Marie Curie 2-year fellowship. M.K. and S.L. were funded by a Boehringer Ingelheim fellowship and an EMBO Long-Term Fellowship, respectively. S.W. is funded by a Wellcome Trust Senior Research Fellowship in the Basic Biomedical Sciences and by funds from the Gatsby Charitable Foundation and Oxford Martin School.

Received: September 25, 2014
Revised: December 8, 2014
Accepted: January 15, 2015
Published: February 26, 2015

References

1. Schultz, W. (2010). Subjective neuronal coding of reward: temporal value discounting and risk. Eur. J. Neurosci. *31*, 2124–2135.
2. Bromberg-Martin, E.S., Matsumoto, M., and Hikosaka, O. (2010). Dopamine in motivational control: rewarding, aversive, and alerting. Neuron *68*, 815–834.
3. Liu, C., Plaçais, P.Y., Yamagata, N., Pfeiffer, B.D., Aso, Y., Friedrich, A.B., Siwanowicz, I., Rubin, G.M., Preat, T., and Tanimoto, H. (2012). A subset of dopamine neurons signals reward for odour memory in Drosophila. Nature *488*, 512–516.
4. Burke, C.J., Huetteroth, W., Owald, D., Perisse, E., Krashes, M.J., Das, G., Gohl, D., Silies, M., Certel, S., and Waddell, S. (2012). Layered reward signalling through octopamine and dopamine in Drosophila. Nature *492*, 433–437.
5. Lin, S., Owald, D., Chandra, V., Talbot, C., Huetteroth, W., and Waddell, S. (2014). Neural correlates of water reward in thirsty Drosophila. Nat. Neurosci. *17*, 1536–1542.
6. Han, K.A., Millar, N.S., and Davis, R.L. (1998). A novel octopamine receptor with preferential expression in Drosophila mushroom bodies. J. Neurosci. *18*, 3650–3658.
7. Burke, C.J., and Waddell, S. (2011). Remembering nutrient quality of sugar in Drosophila. Curr. Biol. *21*, 746–750.
8. Rohwedder, A., Pfitzenmaier, J.E., Ramsperger, N., Apostolopoulou, A.A., Widmann, A., and Thum, A.S. (2012). Nutritional value-dependent and nutritional value-independent effects on Drosophila melanogaster larval behavior. Chem. Senses *37*, 711–721.
9. Selcho, M., Pauls, D., Huser, A., Stocker, R.F., and Thum, A.S. (2014). Characterization of the octopaminergic and tyraminergic neurons in the central brain of Drosophila larvae. J. Comp. Neurol. *522*, 3485–3500.
10. Das, G., Klappenbach, M., Vrontou, E., Perisse, E., Clark, C.M., Burke, C.J., and Waddell, S. (2014). Drosophila learn opposing components of a compound food stimulus. Curr. Biol. *24*, 1723–1730.
11. Schwaerzel, M., Monastirioti, M., Scholz, H., Friggi-Grelin, F., Birman, S., and Heisenberg, M. (2003). Dopamine and octopamine differentiate between aversive and appetitive olfactory memories in Drosophila. J. Neurosci. *23*, 10495–10502.
12. Tanaka, N.K., Tanimoto, H., and Ito, K. (2008). Neuronal assemblies of the Drosophila mushroom body. J. Comp. Neurol. *508*, 711–755.
13. Jenett, A., Rubin, G.M., Ngo, T.T., Shepherd, D., Murphy, C., Dionne, H., Pfeiffer, B.D., Cavallaro, A., Hall, D., Jeter, J., et al. (2012). A GAL4-driver line resource for Drosophila neurobiology. Cell Rep. *2*, 991–1001.
14. Dietzl, G., Chen, D., Schnorrer, F., Su, K.C., Barinova, Y., Fellner, M., Gasser, B., Kinsey, K., Oppel, S., Scheiblauer, S., et al. (2007). A genome-wide transgenic RNAi library for conditional gene inactivation in Drosophila. Nature *448*, 151–156.
15. Kitamoto, T. (2001). Conditional modification of behavior in Drosophila by targeted expression of a temperature-sensitive shibire allele in defined neurons. J. Neurobiol. *47*, 81–92.
16. Hamada, F.N., Rosenzweig, M., Kang, K., Pulver, S.R., Ghezzi, A., Jegla, T.J., and Garrity, P.A. (2008). An internal thermal sensor controlling temperature preference in Drosophila. Nature *454*, 217–220.

17. Lee, T., and Luo, L. (1999). Mosaic analysis with a repressible cell marker for studies of gene function in neuronal morphogenesis. Neuron *22*, 451–461.
18. Perisse, E., Yin, Y., Lin, A.C., Lin, S., Huetteroth, W., and Waddell, S. (2013). Different kenyon cell populations drive learned approach and avoidance in Drosophila. Neuron *79*, 945–956.
19. Pitman, J.L., Huetteroth, W., Burke, C.J., Krashes, M.J., Lai, S.L., Lee, T., and Waddell, S. (2011). A pair of inhibitory neurons are required to sustain labile memory in the Drosophila mushroom body. Curr. Biol. *21*, 855–861.
20. Isabel, G., Pascual, A., and Preat, T. (2004). Exclusive consolidated memory phases in Drosophila. Science *304*, 1024–1027.
21. Yu, D., Akalal, D.B., and Davis, R.L. (2006). Drosophila alpha/beta mushroom body neurons form a branch-specific, long-term cellular memory trace after spaced olfactory conditioning. Neuron *52*, 845–855.
22. Krashes, M.J., and Waddell, S. (2008). Rapid consolidation to a radish and protein synthesis-dependent long-term memory after single-session appetitive olfactory conditioning in Drosophila. J. Neurosci. *28*, 3103–3113.
23. Trannoy, S., Redt-Clouet, C., Dura, J.M., and Preat, T. (2011). Parallel processing of appetitive short- and long-term memories in Drosophila. Curr. Biol. *21*, 1647–1653.
24. Hirano, Y., Masuda, T., Naganos, S., Matsuno, M., Ueno, K., Miyashita, T., Horiuchi, J., and Saitoe, M. (2013). Fasting launches CRTC to facilitate long-term memory formation in Drosophila. Science *339*, 443–446.
25. Sekeres, M.J., Mercaldo, V., Richards, B., Sargin, D., Mahadevan, V., Woodin, M.A., Frankland, P.W., and Josselyn, S.A. (2012). Increasing CRTC1 function in the dentate gyrus during memory formation or reactivation increases memory strength without compromising memory quality. J. Neurosci. *32*, 17857–17868.
26. Monastirioti, M., Linn, C.E.J., Jr., and White, K. (1996). Characterization of Drosophila tyramine beta-hydroxylase gene and isolation of mutant flies lacking octopamine. J. Neurosci. *16*, 3900–3911.
27. Shearin, H.K., Macdonald, I.S., Spector, L.P., and Stowers, R.S. (2014). Hexameric GFP and mCherry reporters for the Drosophila GAL4, Q, and LexA transcription systems. Genetics *196*, 951–960.
28. Gohl, D.M., Silies, M.A., Gao, X.J., Bhalerao, S., Luongo, F.J., Lin, C.C., Potter, C.J., and Clandinin, T.R. (2011). A versatile in vivo system for directed dissection of gene expression patterns. Nat. Methods *8*, 231–237.
29. Shang, Y., Griffith, L.C., and Rosbash, M. (2008). Light-arousal and circadian photoreception circuits intersect at the large PDF cells of the Drosophila brain. Proc. Natl. Acad. Sci. USA *105*, 19587–19594.
30. Thistle, R., Cameron, P., Ghorayshi, A., Dennison, L., and Scott, K. (2012). Contact chemoreceptors mediate male-male repulsion and male-female attraction during Drosophila courtship. Cell *149*, 1140–1151.
31. Sitaraman, D., Zars, M., Laferriere, H., Chen, Y.C., Sable-Smith, A., Kitamoto, T., Rottinghaus, G.E., and Zars, T. (2008). Serotonin is necessary for place memory in Drosophila. Proc. Natl. Acad. Sci. USA *105*, 5579–5584.
32. Nicolaï, L.J., Ramaekers, A., Raemaekers, T., Drozdzecki, A., Mauss, A.S., Yan, J., Landgraf, M., Annaert, W., and Hassan, B.A. (2010). Genetically encoded dendritic marker sheds light on neuronal connectivity in Drosophila. Proc. Natl. Acad. Sci. USA *107*, 20553–20558.
33. Owald, D., Fouquet, W., Schmidt, M., Wichmann, C., Mertel, S., Depner, H., Christiansen, F., Zube, C., Quentin, C., Körner, J., et al. (2010). A Syd-1 homologue regulates pre- and postsynaptic maturation in Drosophila. J. Cell Biol. *188*, 565–579.

Current Biology

Supplemental Information

Sweet Taste and Nutrient Value Subdivide Rewarding Dopaminergic Neurons in *Drosophila*

Wolf Huetteroth, Emmanuel Perisse, Suewei Lin, Martín Klappenbach, Christopher Burke, and Scott Waddell

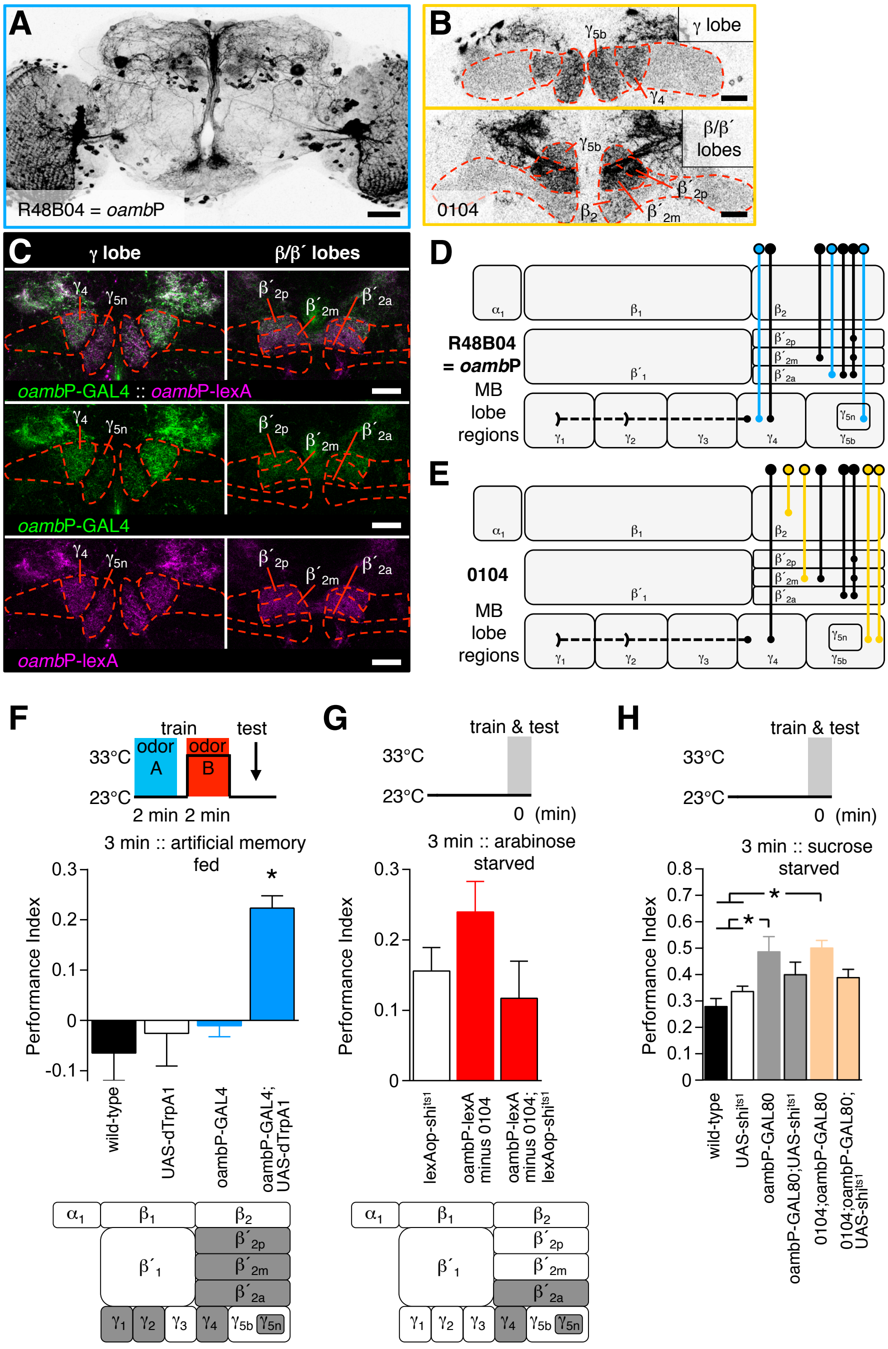

Figure S1. R48B04 (*oamb*P-GAL4, -LexA) and 0104 express in common and unique neurons. Expression data and control experiments related to Figure 1.

(A) Projection view of a confocal stack of a brain from a *oamb*P-GAL4;UAS-mCD8::GFP fly. Scale bar 50 μm. **(B)** Individual confocal sections of 1.5 μm from a 0104-GAL4;UAS-mCD8::GFP brain at the level of the γ or β and β´ lobes, scale bar 20 μm. 0104-GAL4 driven UAS-mCD8::GFP labels ~55 mostly dopaminergic PAM cells [4]. The pattern largely overlaps with that of *oamb*P-GAL4 (Fig. 2A-D), but

0104-GAL4 labels fewer neurons innervating γ_4 and β'_{2a}. 0104-GAL4 also labels neurons innervating β'_{2m} and β_2, plus the γ_{5b} cell type that broadly innervates γ_5. Detail provided in Fig. 2F, G and S3C and is summarized in Fig. S1E. **(C)** *oamb*P-GAL4 driven UAS-mCD8::GFP (green) and *oamb*P-LexA driven lexAop-rCD2::RFP (magenta) label the same subset of about 70 neurons within the PAM cluster. Individual confocal sections of 0.4 µm at the level of the γ or β/β' lobes, scale bar 20 µm. Individual channels and the merge are shown. **(D)** Pictorial of the horizontal lobe innervation by *oamb*P-GAL4 neurons. Blue cells are exclusive to *oamb*P-GAL4, whereas black cells are common to *oamb*P-GAL4 and 0104. The cell type that connects γ_1, γ_2 and γ_4 is previously undescribed and not TH-labeled, so is unlikely to be dopaminergic. **(E)** Schematic of the horizontal lobe innervation by 0104-GAL4 neurons. Yellow cells are exclusive to 0104-GAL4, whereas black cells are common to *oamb*P-GAL4 and 0104-GAL4. **(F)** Pairing odor exposure with UAS-*dTrpA1* activation of *oamb*P-GAL4 neurons in fed flies forms appetitive STM that is significantly different to controls 3 min after training ($p < 0.003$, ANOVA, $n \geq 6$). **(G)** Permissive temperature control for Fig. 2E. STM performance of *oamb*P-LexA/lexAop-*shi*^{ts1};0104/UAS-*lexA*^{RNAi} flies was robust and indistinguishable from that of controls ($p > 0.14$, ANOVA, $n = 12$) when hungry flies were trained with arabinose at permissive 23˚C. **(H)** No significant differences were apparent in STM performance when starved *oamb*P-GAL80;0104-GAL4/UAS-*shi*^{ts1} flies were trained and tested at permissive 23°C (compared to controls, $p > 0.16$, ANOVA, $n = 8$). There is, however, a significant difference between wild type and UAS-*shi*^{ts1} and *oamb*P-GAL80;0104 controls ($p< 0.03$, ANOVA, $n = 8$).

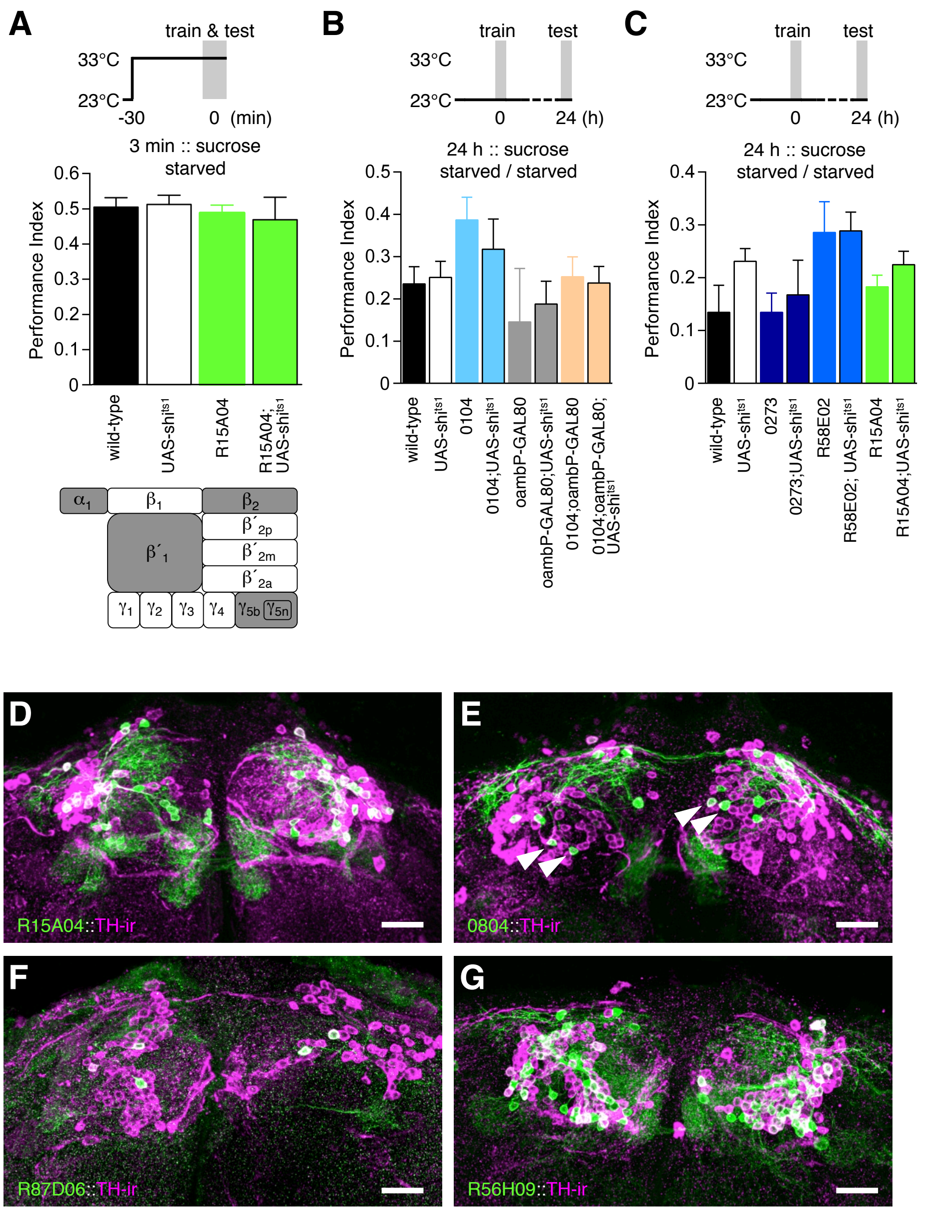

Figure S2. Permissive temperature control experiments and verification of dopaminergic neuron identity for key GAL4 lines used in Figure 2.

(A) Blocking R15A04 neurons with UAS-*shi*^{ts1} expression does not impair STM performance ($p > 0.9$, ANOVA, $n \geq 6$). **(B)** No LTM impairments were evident in starved sucrose-trained 0104-GAL4/UAS-*shi*^{ts1} or *oamb*P-GAL80;0104-GAL4/UAS-*shi*^{ts1} flies when trained and tested at permissive 23˚C ($p > 0.5$, ANOVA, n = 6-8). **(C)** At permissive 23˚C flies expressing UAS-*shi*^{ts1} with 0273, R58E02, or R15A04-GAL4 do not reveal a significant LTM defect following training with sucrose ($p > 0.4$, ANOVA, n = 5-10). **(D)** R15A04 labels ~26 tyrosine hydroxylase (TH)-positive

dopaminergic neurons and ~4 TH-negative neurons in the PAM cluster. TH-ir, anti-TH immunoreactive **(E)** Two of the ~8 neurons labeled by 0804-GAL4 are TH-positive PAM dopaminergic neurons (arrowheads). **(F)** R87D06 labels ~4-8 TH-positive PAM dopaminergic neurons. **(G)** R56H09 labels ~54 neurons in PAM. 34 are TH-positive dopaminergic neurons and the other 20 are non-dopaminergic neurons, representing a new cell type connecting γ_1, γ_2 and γ_4. Scale bars, 20 μm in D-G.

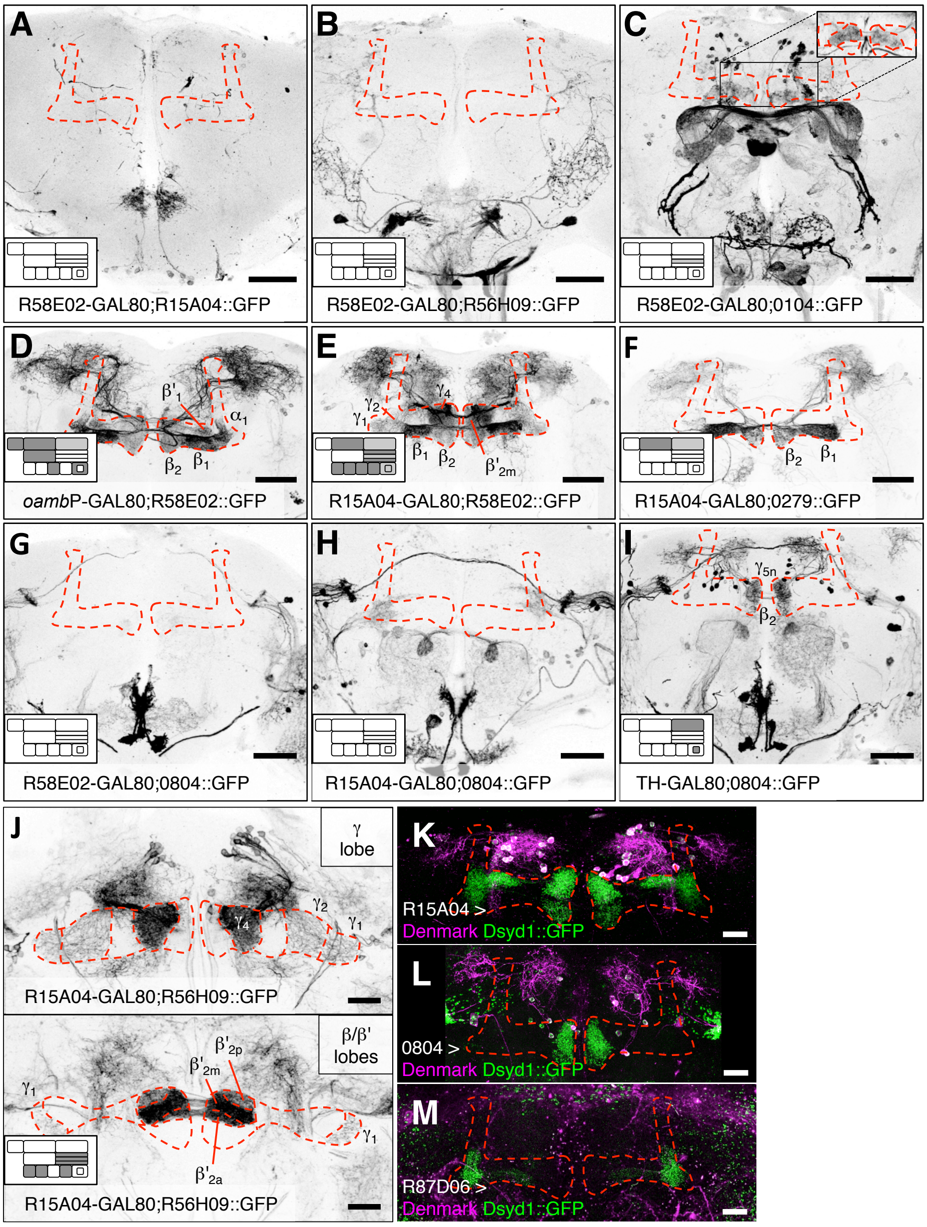

Figure S3. Genetic intersection reveals the relatedness of the dopaminergic neuron-expressing GAL4 lines used in Figure 3 and 4.

(A) R58E02-GAL80 suppresses expression in all R15A04-GAL4 labeled PAM neurons. **(B)** R58E02-GAL80 inhibits expression in R56H09-GAL4 labeled PAM neurons. **(C)** Weak expression remains in ~15 neurons that innervate β'_{2m} region when R58E02-GAL80 is combined with 0104-GAL4. Inset shows a 10 μm sub-projection at the level of the β'_{2} lobe. Scale bar, 50 μm and applies to A-C. **(D)**

*oamb*P-GAL80 combined with R58E02-GAL4;UAS-mCD8::GFP retains expression in neurons innervating the α_1, β'_1, β'_2, β_1, β_2, γ_3 and γ_{5b} regions. No label remains in γ_1, γ_2 or γ_4, and expression is weakened in γ_5 and β'_2. **(E)** R15A04-GAL80 suppresses all expression in α_1 and β'_1 innervating neurons when combined with R58E02-GAL4 driven GFP and weakens labeling in β_2 and γ_5. **(F)** R15A04-GAL80 removes labeling of ~4 somata and reduces innervation in β_2 when combined with 0279-GAL4;UAS-mCD8::GFP. Scale bar, 50 µm and applies to D-F. **(G)** R58E02-GAL80 suppressed all 0804-GAL4;UAS-mCD8::GFP expression in PAM neurons. **(H)** R15A04-GAL80 removed all expression in PAM neurons in 0804-GAL4;UAS-mCD8::GFP. **(I)** *TH*-GAL80 left expression of all ~eight 0804-GAL4 labeled PAM neurons intact, showing that γ_{5n} neurons are not covered by *TH*-GAL80. Scale bar, 50 µm and applies to G-I. **(J)** R15A04-GAL80 combined with R56H09-GAL4;UAS-mCD8::GFP completely suppresses expression in γ_{5n}. The new cell type in γ_1, γ_2 and γ_4 remains, as does strong expression in β'_{2am} and weaker labeling in β'_{2p}. Projection views of 25 and 15 individual 1 µm confocal sections at the level of the γ and β/β' lobes, respectively. Scale bar 20 µm. **(K)** Expression of UAS-DenMark (magenta) and UAS-Dsyd1::GFP (green) reveals that all PAM neuron innervation of the horizontal lobes in R15A04-GAL4 flies is presynaptic. **(L)** DenMark (magenta) and Dsyd1::GFP (green) reveals that all 0804-GAL4 expressing PAM neurons are presynaptic in the horizontal lobes. **(M)** R87D06-GAL4 driven DenMark (magenta) and Dsyd1::GFP (green) reveals that all PAM neurons are presynaptic in the horizontal lobes. Scale bars in K-M 20 µm.

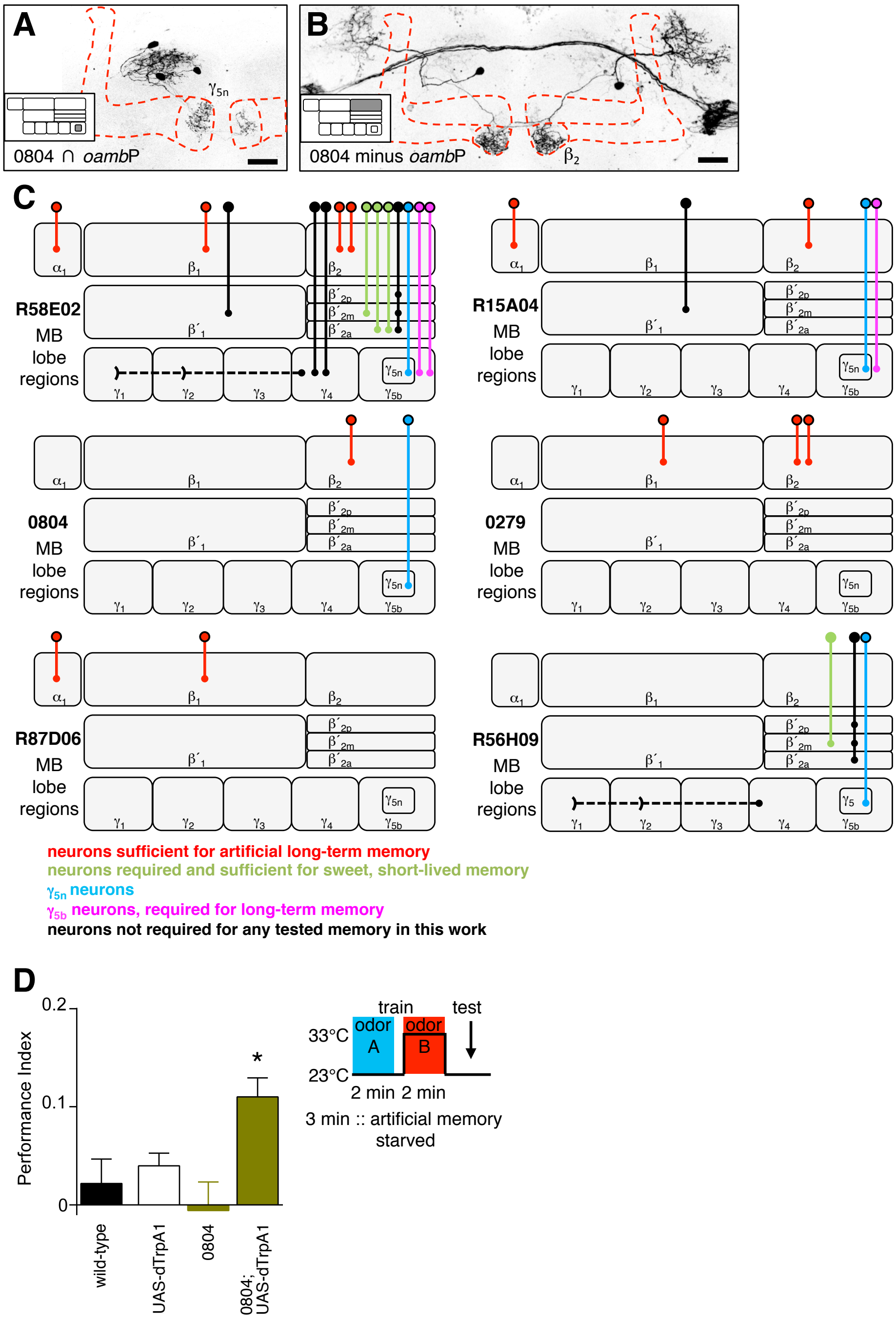

Figure S4. Anatomical detail of dopaminergic neurons innervating the horizontal mushroom body lobes. Data related to Figures 1-4.

(A) Positive genetic intersection between 0804-GAL4 and *oamb*P-LexA using lexAop-FLP in combination with UAS>STOP>GFP reveals mosaic unilateral labeling of three γ_{5n} neurons that innervate a narrow band in the ipsilateral γ_5 and form a commissure to an even smaller innervation in the contralateral γ_5. **(B)** Subtracting *oamb*P-LexA neurons from 0804;UAS-mCD8::GFP expression using lexAop-GAL80

reveals 2 cells innervating only the β_2 zones. Since *oamb*P-GAL4-driven UAS-*dTrpA1* cannot implant LTM whereas 0804;UAS-*dTrpA1* can form robust LTM, these two neurons could be those responsible for reinforcing appetitive LTM (Fig. 3A). Scale bars in A, B 20 µm. **(C)** Schematics of the horizontal mushroom body lobes and the respective regions innervated by R58E02, R15A04, 0804, 0279, R87D06, and R56H09. Red labeled cells are sufficient to implant artificial LTM, green labeled cells are required for sweet-taste reinforced STM. The blue γ_{5n} neuron type is found in all lines except 0279 and R87D06. The magenta cell type γ_{5b} is essential for appetitive LTM formation. The black neurons were apparently not critical for any of the behavioral phenomena in this study. **(D)** Artificially implanted memory using 0804-GAL4 driven UAS-*dTrpA1* can guide behavior immediately after training in starved flies ($p < 0.001$, ANOVA, $n \geq 11$), consistent with residual STM performance in *Tbh*^{M18} mutant flies (Fig. 1A).

dense (black) or partial (grey) coverage in respective region
sufficient for artificial long-term memory
required and sufficient for sweet, short-lived memory
required for long-term memory

	0273	R58E02	R15A04	0104	0104 minus R48B04	R48B04 =*oambP*	R48B04 minus 0104	0279	0804	R87D06	R56H09	TH*	NP1528* NP5272*
α_1	●	●	●							●			
β_1	●	●						●		● (grey)			
β_{2sc}	●	●	● (grey)	●				●	● (grey)				
β_{2s}	●											●	●
β'_1	●	●	●										
β'_{2a}	●	●		● (grey)		●	● (grey)				● (grey)		
β'_{2m}	●	● (grey)		●	● (grey)	● (grey)					● (grey)		
β'_{2p}	●	●		● (grey)		● (grey)					● (grey)		
γ_1												●	
γ_2												●	
γ_3	●	●											
γ_4	●	●		● (grey)		●	● (grey)						
γ_{5b}	●	●	● (grey)	●	●							● (grey)	
γ_{5n}	●	●	●			●	●		●		●		
MB-M9	●	●		●		●					●		

* TH-GAL4 expression according to Aso et al. 2012, modified

Supplementary Table 1. Overview of dopaminergic neurons labeled in each GAL4 line detailing their zonal innervation within the mushroom body lobes. Black dots represent coverage of the respective line in that region; grey dots represent partial coverage. Red background denotes regions which can form artificial LTM, if stimulation of their respective dopaminergic neurons is paired with odor exposure. Green background marks regions innervated by neurons that are required for sweet-taste reinforced STM. Magenta background marks the γ_{5b} cell type that is required for nutrient LTM formation. Innervation information for TH-GAL4 and the MB-M3 lines NP1528 and NP5272, that can mediate aversive reinforcement, is taken

from previous studies [17, 33, 34]. Abbreviations: α_1, β_1, β'_1, γ_1, γ_2, γ_3, γ_4, and γ_5: regions of the basal vertical α lobe, horizontal β, β', and γ lobe respectively; β_{2sc}: dopaminergic neuron type covering both core and surface in β_2; β_{2s}: surface region only in the β lobe tip, covered by dopaminergic neuron type MB-M3; β'_{2a}: anterior part of the β'lobe tip; β'_{2m}: median part of the β' lobe tip; β'_{2p}: dorsoposterior part of the β'lobe tip; γ_{5b}: broad innervation of γ_5, the distal γ lobe tip; γ_{5n}: cell type that leads to a narrow-banded innervation pattern of the γ lobe tip. MB-M9: new PAM cell-type that covers g_1, g_2, and g_4 and is not labeled with anti-TH antibody.

Additional References:

34. Aso, Y., Herb, A., Ogueta, M., Siwanowicz, I., Templier, T., Friedrich, A.B., Ito, K., Scholz, H., and Tanimoto, H. (2012). Three dopamine pathways induce aversive odor memories with different stability. PLoS Genet. 8, e1002768
